# Forming next-generation antibody–nanoparticle conjugates through the oriented installation of non-engineered antibody fragments†
†Electronic supplementary information (ESI) available. See DOI: 10.1039/c7sc02747h


**DOI:** 10.1039/c7sc02747h

**Published:** 2017-08-14

**Authors:** Michelle K. Greene, Daniel A. Richards, João C. F. Nogueira, Katrina Campbell, Peter Smyth, Marcos Fernández, Christopher J. Scott, Vijay Chudasama

**Affiliations:** a Centre for Cancer Research and Cell Biology , School of Medicine , Dentistry and Biomedical Sciences , Queen's University Belfast , Belfast , UK . Email: c.scott@qub.ac.uk; b Department of Chemistry , University College London , London , UK . Email: v.chudasama@ucl.ac.uk; c Institute for Global Food Security , School of Biological Sciences , Queen's University Belfast , Belfast , UK; d Research Institute for Medicines (iMed.ULisboa) , Faculty of Pharmacy , Universidade de Lisboa , Lisbon , Portugal

## Abstract

The successful development of targeted nanotherapeutics is contingent upon the conjugation of therapeutic nanoparticles to target-specific ligands, with particular emphasis being placed on antibody-based ligands. Thus, new methods that enable the covalent and precise installation of targeting antibodies to nanoparticle surfaces are greatly desired, especially those which do not rely on costly and time-consuming antibody engineering techniques. Herein we present a novel method for the highly controlled and oriented covalent conjugation of non-engineered antibody F(ab) fragments to PLGA–PEG nanoparticles using disulfide-selective pyridazinedione linkers and strain-promoted alkyne–azide click chemistry. Exemplification of this method with trastuzumab and cetuximab showed significant improvements in both conjugation efficiency and antigen binding capability, when compared to commonly employed strategies for antibody–nanoparticle construction. This new approach paves the way for the development of antibody-targeted nanomedicines with improved paratope availability, reproducibility and uniformity to enhance both biological activity and ease of manufacture.

## Abstract 2

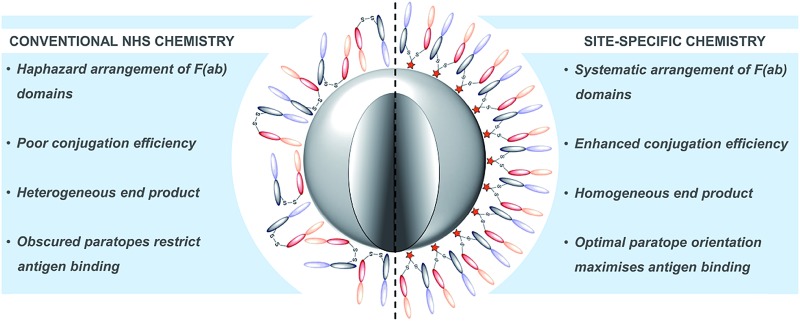
Enabling oriented installation of non-engineered antibody fragments on nanoparticle surfaces to create next-generation antibody–nanoparticle conjugates.

## Introduction

The application of nanoparticles as drug delivery vehicles has attracted significant interest owing to potential benefits of these platforms, such as improved pharmacokinetic and safety profiles of encapsulated cargo. Several nanoformulations are now marketed, whilst numerous others are under clinical evaluation.^1^,^2^ Many nanoformulations are developed for oncology applications in particular.^3^ Nanoparticles can ‘passively’ accumulate within tumours by exploiting defects in neovasculature endothelial junctions and impaired lymphatic drainage, a phenomenon known as the enhanced permeability and retention (EPR) effect. However, surface functionalisation of nanoparticles with targeting ligands has the potential to significantly enhance cellular uptake and retention at the tumour site in a concept referred to as ‘active’ targeting.^4^–^6^

A variety of ligands have been explored for such purposes, including aptamers, peptides and carbohydrates, although antibodies are perhaps the most frequently employed.^7^–^10^ Numerous bioconjugation methods exist to graft these ligands to the surface of nanoparticles, with common approaches including carbodiimide and maleimide chemistries.^11^,^12^ Carbodiimide coupling involves the derivatisation of a carboxyl group with cross-linking agents such as 1-ethyl-3-(-3-dimethylaminopropyl) carbodiimide (EDC), followed by their direct conjugation to amines resulting in the generation of an amide bond. Depending on the direction of the conjugation, antibodies can be coupled to nanoparticles by virtue of free amine or carboxyl functionalities on lysine or aspartic/glutamic acid residues, respectively. However, this approach is encumbered by low reaction efficiencies and generates highly heterogeneous nanoconjugates, where optimal orientation and functionality of paratopes cannot be guaranteed due to the high abundance of reactive amine- and carboxyl-containing residues throughout antibodies. Alternatively, maleimide chemistry can in theory facilitate site-specific conjugation to cysteine residues liberated by the reduction of the inter-strand disulfide bonds of antibodies.^12^–^14^ However, multiple reports have questioned the cysteine selectivity of maleimide conjugation under commonly employed conditions,^15^,^16^ and the resultant bioconjugate would bear a thioether bond that has been shown to be inherently unstable *in vivo*.^17^–^19^ Furthermore this strategy results in the loss of a covalent link between antibody chains. Nonetheless, reports throughout the literature demonstrate the advantages of utilising other site-specific chemistries for the generation of nanoconjugates, including improved antigen binding and greater product homogeneity.^9^,^12^,^20^,^21^ Many of these approaches involve the installation of site-selective amino acid residues using site-directed mutagenesis, followed by subsequent pairing with an appropriate reactive group on the nanoparticle surface.^9^,^14^,^22^–^26^ Whilst this approach allows for the oriented presentation of antibody ligands, it is restricted by the need for time consuming and expensive antibody engineering.

**Fig. 1:**
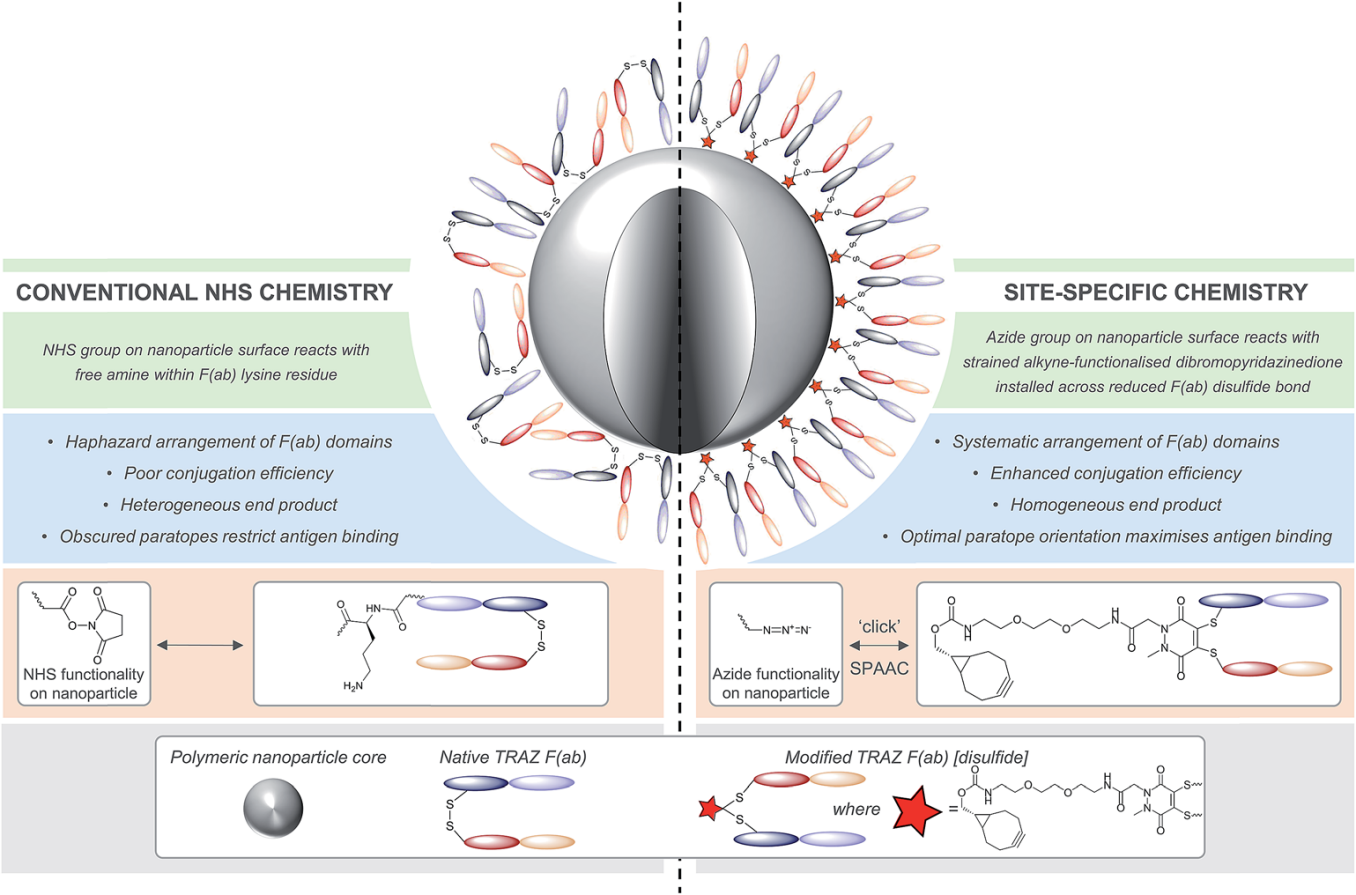
Comparison of site-specific conjugation approach *versus* conventional NHS coupling. Use of a novel heterobifunctional linker permits the controlled assembly of a surface corona of F(ab) targeting ligands on nanoparticles. The concentrated display of oriented and accessible paratopes afforded by this approach maximises target interactions, leading to significantly enhanced avidity *versus* conventional coupling chemistries.

Given these challenges, novel strategies are required to refine antibody conjugation to nanoparticles, with emphasis on: (i) optimising the presentation of the antibody for maximal interaction with cognate targets; and (ii) improving the homogeneity and efficiency of conjugation of antibodies in their native state to aid manufacturability. Previously, we described a novel approach for the functional re-bridging of native inter-strand disulfide bonds of full antibodies and their constituent fragments, *i.e.* resulting in no loss of covalent linkage between antibody chains and modifying at positions that are distal from the antibody binding site.^27^–^33^ This involved the selective insertion of pyridazinedione moieties bearing reactive handles into reduced disulfide bonds, thus enabling site-specific incorporation of ‘click’ domains without impinging upon antibody functionality. Here we describe the application of this disulfide re-bridging technology to site-selectively modify trastuzumab (TRAZ) F(ab) to bear a strained alkyne handle distal to the paratope and conjugate it to azide-functionalised nanoparticles. This novel approach is compared to conventional methods in order to determine the importance of chemical strategy on the performance of nanoconjugates (Fig. 1). We demonstrate superior binding to HER2 of nanoconjugates formed by our new method *versus* NHS ester conjugation, highlighting the potential of this approach to overcome the shortcomings of conventional coupling chemistries employed in many targeted nanoformulations to date.

## Results and discussion

**Scheme 1:**
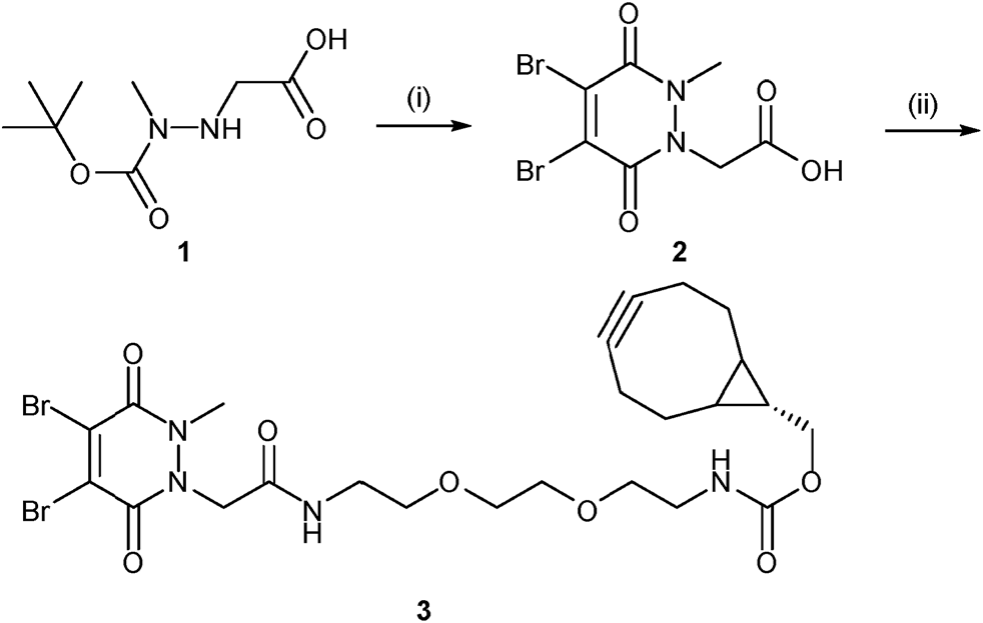
Synthesis of strained alkyne functionalised pyridazinedione **3**. Reagents and conditions: (i) dibromomaleic acid, AcOH, reflux, 24 h; (ii) PyBOP, DIPEA, CH_2_Cl_2_, 21 °C, 16 h.

Our study began with the synthesis of a novel heterobifunctional linker that would facilitate conjugation to an antibody disulfide at one end and attachment to a nanoparticle at the other. For attachment to the nanoparticle surface, we decided to incorporate the strained alkyne BCN due to its ability to engage in copper free strained-promoted alkyl-azide cycloaddition (SPAAC) reactions and our confidence in being able to formulate azide-functionalised nanoparticles (discussed in detail below). Despite wide-spread use within biomedical research, SPAAC has remained largely unexplored for the generation of nanoconjugates and we took this opportunity to appraise it in this light. To impart site-selective protein reactivity to the linker, the BCN moiety was linked to a dibromopyridazinedione, chosen for its exquisite disulfide reactive selectivity and the excellent stability profile of antibody conjugates formed thereof. Synthesis of the strained alkyne functionalised pyridazinedione **3** proceeded from readily available starting materials in a facile manner over three steps (Scheme 1, further details on the synthetic methods can be found in S1–S5 in the ESI†).

**Fig. 2:**
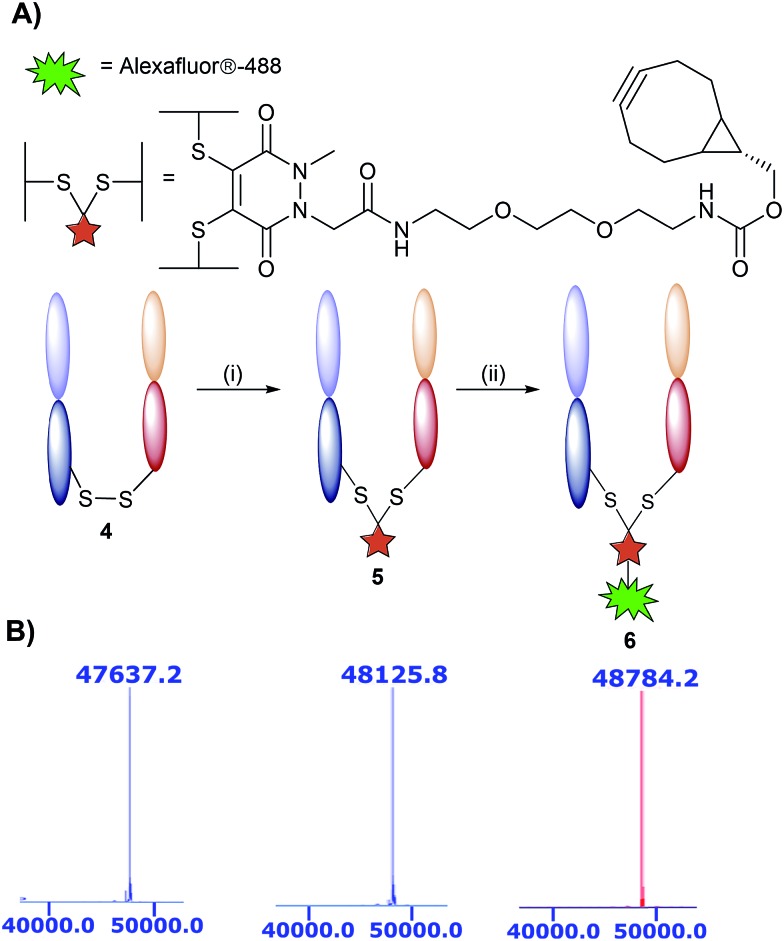
Modification of TRAZ F(ab) with strained alkyne functionalised pyridazinedione **3**. (A) Scheme 2: reagents and conditions: (i) **3**, TCEP·HCl, BBS pH = 8.0 (2 mM EDTA), 4 °C, 15 h; (ii) Alexafluor®-488-N_3_ BBS pH = 8.0 (2 mM EDTA). (B) LCMS data showing native **4**, conjugated **5**, and clicked TRAZ F(ab) **6**. Spectra have been annotated for clarity, see Fig. S7, S9 and S12 in the ESI† for full spectral data.

The monoclonal antibody TRAZ was chosen as a targeting ligand due to its clinical relevance as an approved therapeutic against HER2+ breast cancers.^34^ The choice to utilise the F(ab) domain of TRAZ as the targeting component was driven primarily by the growing volume of evidence suggesting antibody fragments provide multiple benefits to the overall performance of nanoconjugates when compared to full antibodies; F(ab)s retain the binding component of a full antibody.^9^ Additionally, the use of the F(ab) domain is ideal in that it only contains a single solvent accessible disulfide bond to ensure homogeneous modification and that there is only a single site from which the antibody ligand can be attached to the nanoparticle. Furthermore, F(ab) domains can be readily expressed and/or obtained from native full antibody scaffolds *via* simple enzymatic digestion procedures. In this particular case, we obtained TRAZ F(ab) from native TRAZ *via* enzymatic digestion (pepsin followed by papain, further details provided in the ESI†). Site-selective modification of TRAZ F(ab) with strained alkyne-pyridazinedione **3** was achieved according to previously reported protocols^31^ (Fig. 2A) and confirmed using LCMS (Fig. 2B and S9†), and SDS-PAGE showed no aggregation or fragmentation (Fig. S8†) (hereafter referred to as modified TRAZ F(ab) [disulfide] **5**). A test click reaction with Alexafluor-488®-azide confirmed the presence of the strained alkyne (Fig. S10 and S11†). As an appropriate control to determine the overall effect of the site-directed chemistry, a strained alkyne was incorporated into TRAZ F(ab) using non-site-selective lysine-NHS chemistry (modified TRAZ F(ab) [lys]) (Fig. S13 and S14†). Additionally, the F(ab) fragment of cetuximab (CTX) was site-selectively modified to provide a control for antigen target specificity (modified CTX F(ab) [disulfide]) (Fig. S17 and S18†). CTX was considered a suitable targeting ligand control in this context, given that it does not engage HER2 but rather binds to EGFR, another member of the ErbB family of receptor tyrosine kinases.^35^

**Fig. 3:**
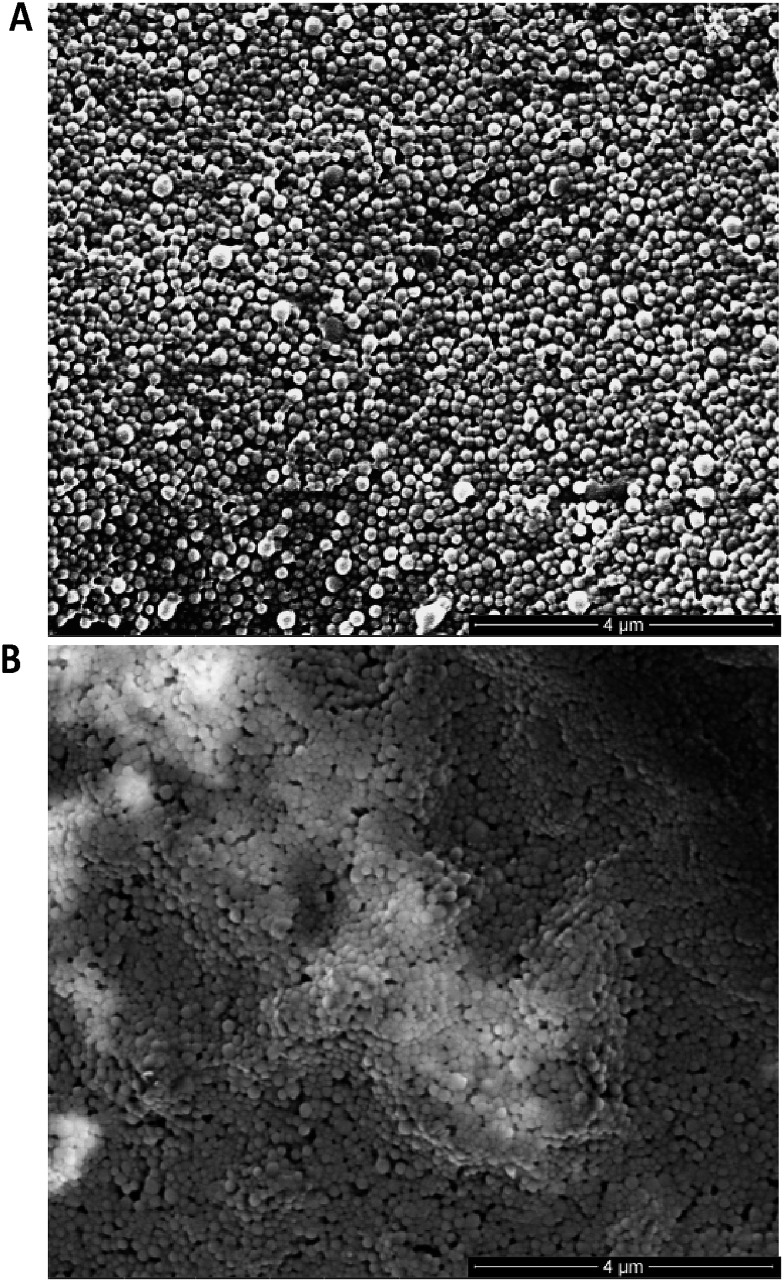
ESEM imaging of nanoformulations. (A) Modified TRAZ F(ab) NP [disulfide] and (B) native TRAZ F(ab) NP were dried on double-sided copper tape mounted onto aluminium stubs, sputter-coated with gold and imaged with an ESEM. Scale bar = 4 μm.

Following preparation of the various F(ab) domains, we next explored the potential for site-specific functionalisation of nanoparticles with modified TRAZ F(ab) [disulfide] **5**. To facilitate ‘click’ conjugation to this antibody fragment, a novel PLGA nanoparticle incorporating a complementary azide moiety was developed. A single emulsion solvent evaporation approach was employed to generate a homogeneous population of azide-terminated nanoparticles from a 25% : 75% polymer blend of PLGA–PEG-azide and PLGA RG502H (nude azide NP) (Table 1). These nanoparticles were subjected to stability assessment over several months, with no significant change in physicochemical characteristics observed upon storage at 4 °C or –20 °C (Fig. S19†). Conjugation of modified TRAZ F(ab) [disulfide] **5** to azide-functionalised nanoparticles was then enabled by incubating both components for 2 h under ambient conditions, yielding a nanoconjugate with a protein loading of 193.1 ± 49.9 pmoles per mg polymer (modified TRAZ F(ab) NP [disulfide]) (Table 1). Contrary to our approach, similar reports of the ‘click’ functionalisation of nanoparticles *via* alkyne–azide cycloaddition most often involve copper catalysis, which can impart toxicities that ultimately limit the biomedical application of the nanoconjugate.^36^–^40^ A range of equimolar controls were also formulated in parallel, which included: (i) native TRAZ F(ab) **4** conjugated to NHS-functionalised nanoparticles (native TRAZ F(ab) NP), (ii) modified TRAZ F(ab) [lys] conjugated to azide-functionalised nanoparticles, with the linker chemistry being introduced at lysine residues as opposed to reduced disulfides (modified TRAZ F(ab) NP [lys]), (iii) native CTX F(ab) conjugated to NHS-functionalised nanoparticles (native CTX F(ab) NP) and (iv) modified CTX F(ab) [disulfide] conjugated to azide-functionalised nanoparticles (modified CTX F(ab) NP [disulfide]) (Table 1). Quantification of F(ab) content within these nanoformulations revealed that conjugation *via* the strained alkyne proceeded with much greater efficiency compared to NHS ester chemistry (Table 1). These findings are consistent with the enhanced reaction kinetics of SPAAC click chemistry over NHS ester chemistry and also the improved stability of the surface bound azide when compared to the activated carboxylic acid. Further characterisation experiments included ESEM imaging, which revealed the spherical morphology and uniform size distribution of selected nanoformulations, with similar diameters to those acquired *via* dynamic light scattering (DLS) in Table 1 (Fig. 3).

**Table 1:** Characterisation of nanoformulations

Nanoformulation	Polymer	Diameter^*a*^ (nm)	PDI^*a*^	Zeta potential^*a*^ (mV)	F(ab) conjugated (pmoles per mg polymer)^*a*^ ^,^^*b*^	Conjugation efficiency^*a*^ (%)
Nude NHS NP	PLGA–PEG-NHS	191.1 ± 1.2	0.16 ± 0.01	–4.6 ± 0.6	—	—
Native TRAZ F(ab) NP	PLGA–PEG-NHS	210.4 ± 2.7	0.18 ± 0.01	–3.5 ± 0.1	65.3 ± 24.0	6.2 ± 2.3
Native CTX F(ab) NP	PLGA–PEG-NHS	191.2 ± 1.8	0.11 ± 0.01	–3.9 ± 0.4	103.8 ± 29.1	9.9 ± 2.8
Nude azide NP	PLGA–PEG-azide	187.4 ± 1.8	0.02 ± 0.01	–2.7 ± 0.6	—	—
Modified TRAZ F(ab) NP [disulfide]	PLGA–PEG-azide	192.4 ± 1.5	0.05 ± 0.02	–1.9 ± 0.7	193.1 ± 49.9	18.4 ± 4.7
Modified TRAZ F(ab) NP [lys]	PLGA–PEG-azide	207.4 ± 0.1	0.13 ± 0.02	–3.1 ± 0.3	475.0 ± 221.7	45.2 ± 21.1
Modified CTX F(ab) NP [disulfide]	PLGA–PEG-azide	189.9 ± 0.2	0.05 ± 0.01	–2.3 ± 0.1	208.4 ± 86.5	19.8 ± 8.2

^*a*^Data expressed as mean ± SD.

^*b*^Equimolar amounts of each F(ab) domain were initially added to the nanoparticle conjugation reaction.

**Fig. 4:**
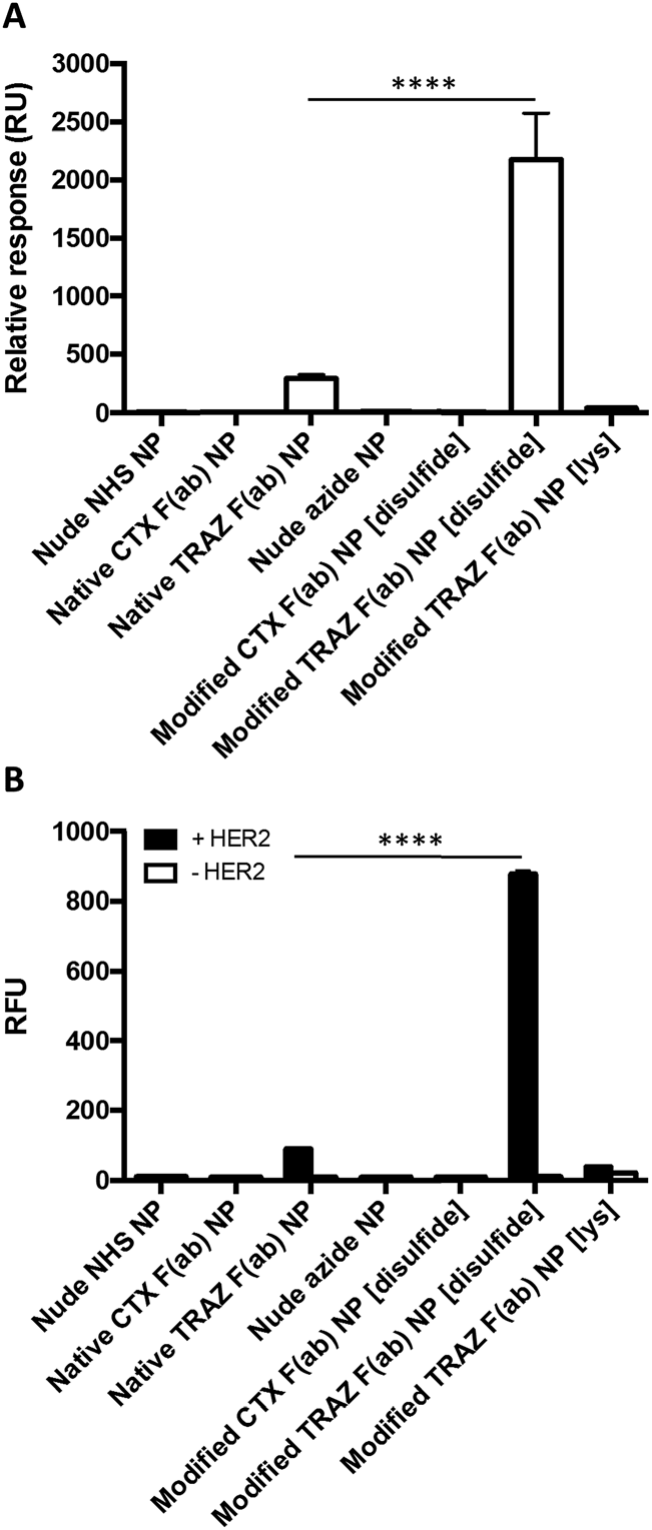
Enhanced binding of modified TRAZ F(ab) NP [disulfide] to HER2 *versus* native TRAZ F(ab) NP. (A) HER2 binding activity of native TRAZ F(ab) NP, modified TRAZ F(ab) NP [disulfide] and associated controls (10 mg polymer per mL) was assessed by SPR. (B) HER2 binding activity of rhodamine 6G-loaded native TRAZ F(ab) NP, modified TRAZ F(ab) NP [disulfide] and associated controls (200 μg polymer per mL) was assessed by modified ELISA. Data expressed as mean ± SEM. Statistical significance was established by one-way ANOVA and Tukey's post-hoc test (****p ≤ 0.0001).

Having successfully developed modified TRAZ F(ab) NP [disulfide], we next explored the ability of the nanoconjugate to bind to the HER2 target receptor. Initial studies employed surface plasmon resonance (SPR) to examine binding activity towards a HER2 fusion protein immobilised on a carboxymethylated dextran chip. Although binding of native TRAZ F(ab) NP was detected *via* SPR, an equivalent polymer concentration of modified TRAZ F(ab) NP [disulfide] showed a significantly enhanced binding profile (Fig. 4A). No appreciable binding of nude NP (both NHS and azide) or CTX F(ab) NP (both native and modified) controls was observed, confirming the dependence of the interaction on TRAZ F(ab). Moreover, HER2 binding of modified TRAZ F(ab) NP [lys] was also negligible despite highly efficient coupling of the fragment to nanoparticles (see Table 1), indicating that this conjugation approach restricted paratope accessibility. This comparison demonstrates that the site-selective nature of pyridazinedione conjugation to F(ab) plays a critical role in the observed improvements in antigen binding; indicating improved paratope accessibility granted by the oriented display of the fragments on the nanoparticle surface.

To exclude the possibility that enhanced binding of modified TRAZ F(ab) NP [disulfide] is a consequence of free F(ab) complexation rather than direct coupling to nanoparticles, we also analysed HER2 binding capacity by modified ELISA. Rhodamine 6G was encapsulated within the various nanoformulations to enable a fluorescent readout of binding to immobilised HER2 fusion protein. These studies produced similar trends to earlier SPR analyses, demonstrating superior binding to HER2 of modified TRAZ F(ab) NP [disulfide] *versus* native TRAZ F(ab) NP and associated controls (Fig. 4B). Consistent with our findings, direct comparisons of carbodiimide and click chemistry-based approaches for nanoparticle functionalisation have also been described in the literature with a similar enhancement in targeting efficiency conferred by the latter.^41^–^45^ Several of these reports employ full antibodies as targeting ligands whereas our approach offers a distinct advantage through the use of F(ab) fragments comprising a sole disulfide bond. This ensures that the click-reactive handle is exclusively installed at a single site located distal from the paratope.

**Fig. 5:**
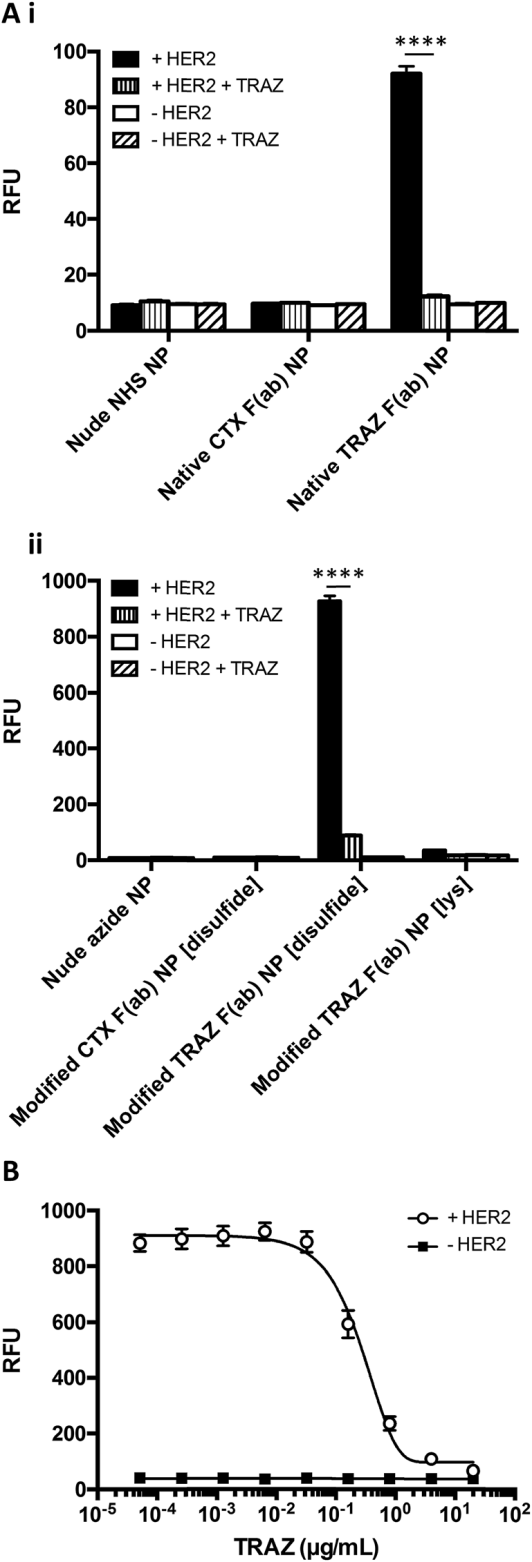
HER2 targeting specificity of TRAZ F(ab) NP. (A) HER2 binding activity of (i) native TRAZ F(ab) NP, (ii) modified TRAZ F(ab) NP [disulfide] and associated controls (200 μg polymer per mL) was assessed by modified ELISA ± pre-block with TRAZ full antibody (20 μg mL^–1^). (B) HER2 binding activity of modified TRAZ F(ab) NP [disulfide] (200 μg polymer per mL) was assessed by modified ELISA in competition with TRAZ full antibody (0.000051–20 μg mL^–1^). Data expressed as mean ± SEM. Statistical significance was established by one-way ANOVA and Tukey's post-hoc test (****p ≤ 0.0001).

The HER2 targeting specificity of modified TRAZ F(ab) NP [disulfide] and native TRAZ F(ab) NP was next validated *via* modified ELISA, where pre-incubation of HER2-coated wells with an excess of TRAZ full antibody significantly impeded nanoparticle binding (Fig. 5Ai and 5Aii). These findings were further bolstered by competition modified ELISA formats, where simultaneous addition of TRAZ full antibody and modified TRAZ F(ab) NP [disulfide] to HER2-coated wells inhibited nanoparticle binding in a concentration-dependent manner (Fig. 5B). Collectively, these results demonstrate the successful coupling of TRAZ F(ab) domains to NHS- and azide-functionalised nanoparticles by distinct approaches, generating active nanoconjugates with retained binding capacity for the cognate HER2 antigen.

**Fig. 6:**
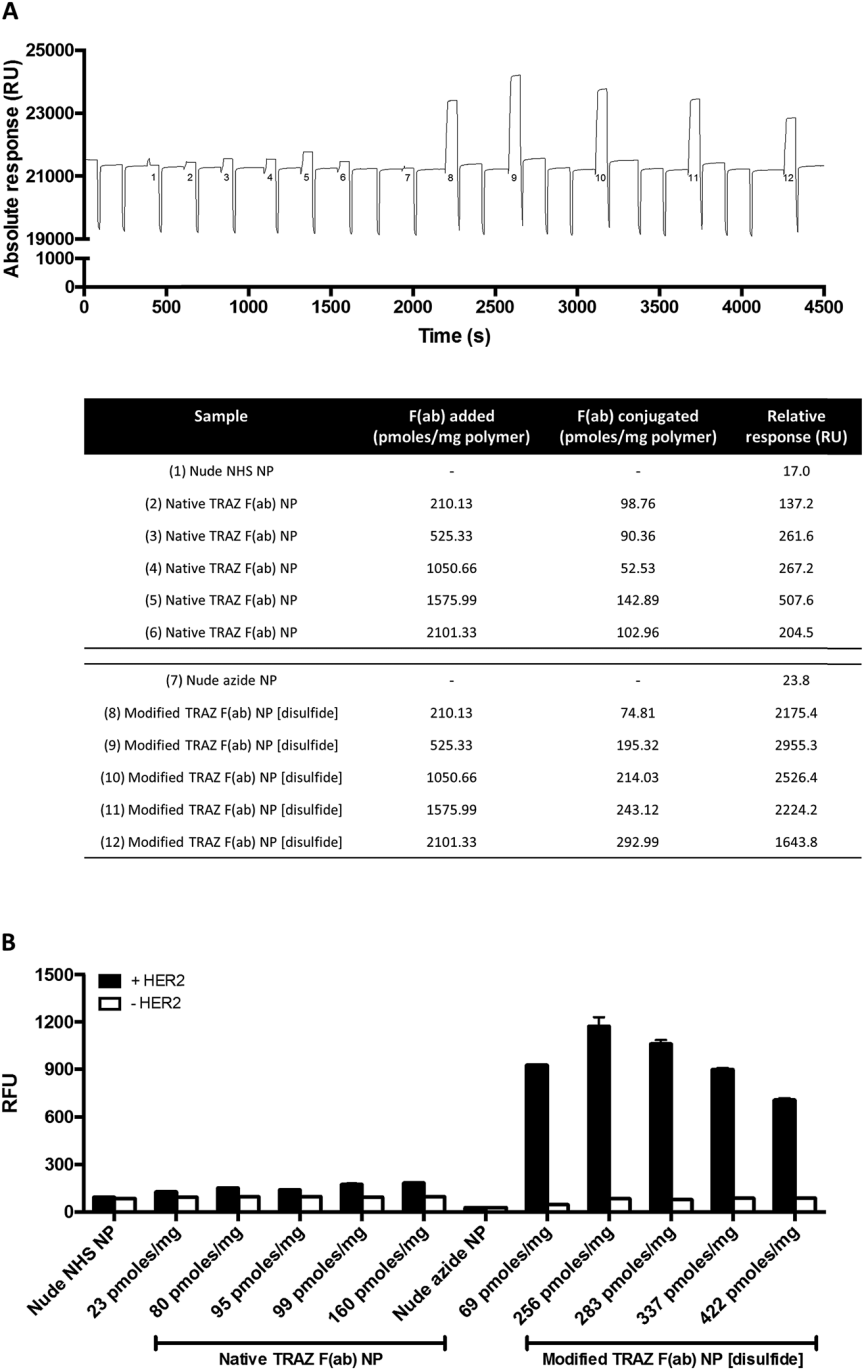
Enhanced binding of modified TRAZ F(ab) NP [disulfide] is not attributed to improved protein conjugation. (A) HER2 binding activity of native TRAZ F(ab) NP or modified TRAZ F(ab) NP [disulfide] (10 mg polymer per mL) with various protein loadings was assessed by SPR. Representative sensorgram shown, with corresponding details for numbered samples in the below table. (B) HER2 binding activity of native TRAZ F(ab) NP or modified TRAZ F(ab) NP [disulfide] (400 μg polymer per mL) with various protein loadings was assessed by modified ELISA. Protein loadings are expressed as pmoles of F(ab) per mg polymer. Data expressed as mean ± SEM.

We next examined the basis for the enhanced HER2 binding activity of the nanoconjugate generated using the site-selective chemistry, modified TRAZ F(ab) NP [disulfide]. To assess whether this effect was simply attributed to enhanced F(ab) loading on the nanoconjugate rather than optimised paratope orientation, we formulated both native TRAZ F(ab) NP and modified TRAZ F(ab) NP [disulfide] using various input amounts of the antibody fragments, ranging from approximately 210 to 2100 pmoles per mg polymer. Higher loadings of modified TRAZ F(ab) [disulfide] **5** on azide-functionalised nanoparticles were observed, with stepwise increases in coupling that correlated with the initial amount of F(ab) added, highlighting the unique degree of control afforded by this conjugation approach (Fig. 6A). As before, enhanced binding of modified TRAZ F(ab) NP [disulfide] was observed upon SPR analysis, even when the F(ab) loading was almost half that of native TRAZ F(ab) NP (Fig. 6A, nanoformulations 5 and 8). This suggests that significant benefits can be achieved even in the case of lower F(ab) loadings, establishing the positive effect of using more controlled (enabling orientation) chemistries. Intriguingly, this data also revealed that HER2 binding activity diminished with higher loadings of modified TRAZ F(ab) [disulfide] **5** on nanoparticles, suggestive of potential steric hindrance effects leading to suboptimal paratope display. Using fluorescently labelled nanoparticles, these studies were replicated *via* modified ELISA, with comparable findings to SPR analyses (Fig. 6B).

**Fig. 7:**
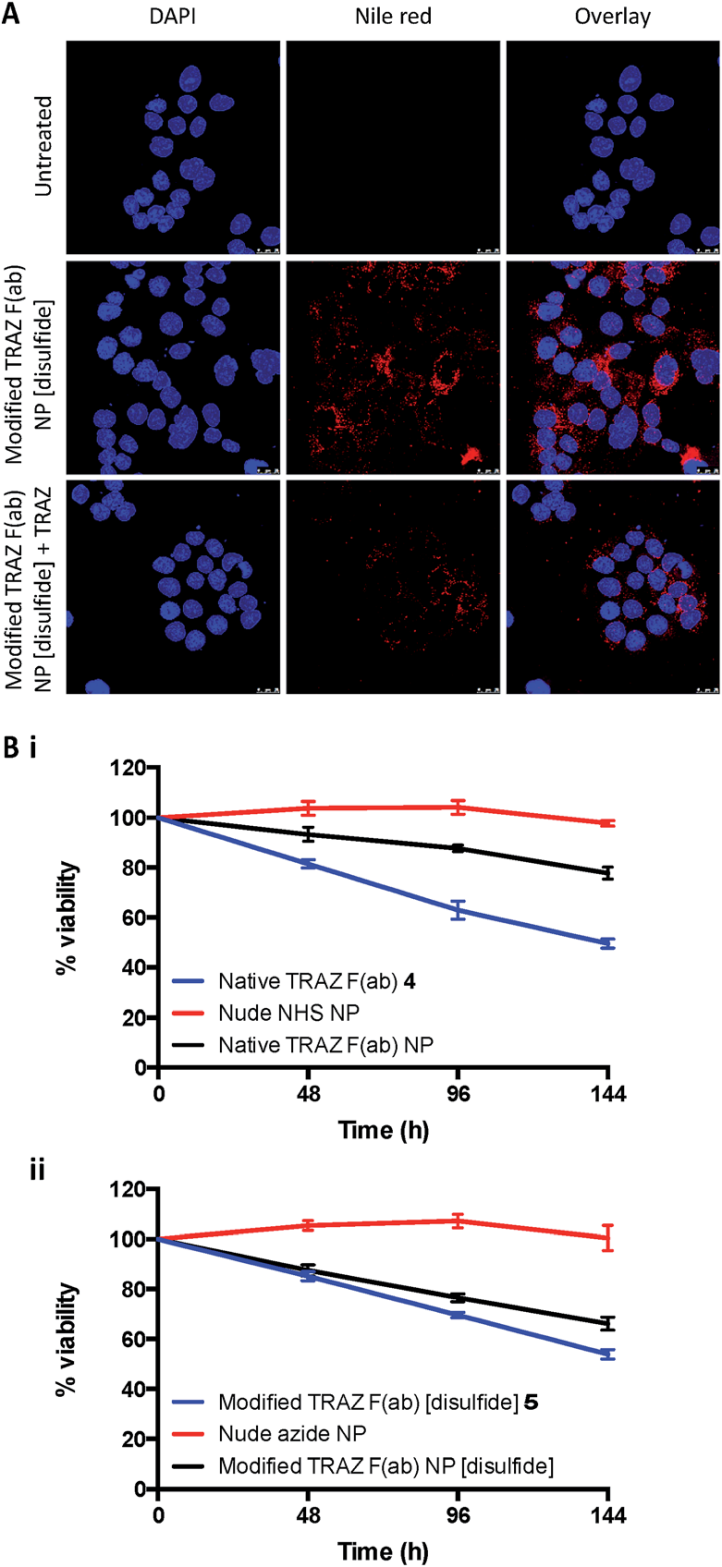
Validation of TRAZ F(ab) functionality in cell-based assays. (A) Confocal microscopy images of HCC1954 cells treated with modified TRAZ F(ab) NP [disulfide] encapsulating nile red (400 μg polymer per mL) ± TRAZ full antibody (200 μg mL^–1^) for 18 h. Blue and red staining denote nuclei and nanoparticles, respectively. Scale bar = 25 μm. (B) BT474 cells were treated with (i) native TRAZ F(ab) NP or (ii) modified TRAZ F(ab) NP [disulfide] and associated controls (500 μg polymer per mL). CellTiter-Glo assay was performed at 48, 96 and 144 h following treatment. Data expressed as mean ± SEM.

Binding of modified TRAZ F(ab) NP [disulfide] to HER2 was then evaluated in a more biologically relevant context using cell-based assays. The nanoconjugate was fluorescently labelled *via* encapsulation of nile red and incubated with the HER2-positive HCC1954 breast cancer line (Fig. S20†), with confocal microscopy demonstrating a clear association of modified TRAZ F(ab) NP [disulfide] with these cells (Fig. 7A). Co-incubation with an excess of TRAZ full antibody markedly ablated fluorescent labeling of the cells, indicating that the binding of the modified TRAZ F(ab) NP [disulfide] was HER2-dependent.

In a final series of studies, we examined the therapeutic effects of the TRAZ F(ab) nanoconjugates *in vitro*. These experiments could not be undertaken with the HCC1954 cell line used for confocal analyses, given that it shows limited sensitivity to TRAZ. However, numerous reports have demonstrated that TRAZ full antibody or its constituent fragments can reduce the viability of BT474 breast cancer cells and so this line was deemed an appropriate model following confirmation of HER2 expression (Fig. S20†).^46^–^49^ Here, we assessed whether TRAZ F(ab) could induce a similar reduction in viability of BT474 cells when presented in a nanoparticle-bound format. Whilst treatment with free native TRAZ F(ab) **4** led to a gradual reduction in cell viability over time as anticipated, this effect was much less pronounced for the corresponding nanoconjugate (Fig. 7Bi). However, upon treatment with modified TRAZ F(ab) NP [disulfide], the reduction in cell viability was much more comparable to free modified TRAZ F(ab) [disulfide] **5** (Fig. 7Bii). These findings are consistent with enhanced paratope accessibility on modified TRAZ F(ab) NP [disulfide] conferred *via* the site-specific conjugation approach. Importantly, these studies also confirmed that the installation of a pyridazinedione linker did not adversely affect the functionality of TRAZ F(ab). Previous work by ourselves and others has shown that antibody display on nanoparticle surfaces can enhance receptor cross-linking; thus mimicking ligand interactions to enhance downstream biological effects.^8^,^50^,^51^ However, as HER2 is a ligand-less receptor, it is not surprising that no enhancement of the effect on cell viability was observed with these particular non-drug loaded nanoconjugates.

To conclude, we have described a novel strategy for the site-specific functionalisation of nanoparticles that promotes the uniform and outward projection of paratopes for maximal target interaction. Using TRAZ F(ab) as a model platform, we demonstrate the successful re-bridging of the inter-chain disulfide bond with a heterobifunctional linker and subsequent ‘click’ coupling to nanoparticles bearing complementary azide moieties. The controlled orientation of TRAZ F(ab) afforded by this approach leads to superior binding to HER2 when compared with conventional NHS ester coupling chemistry, with retention of F(ab) functionality in cell-based assays, demonstrating the importance of controlled chemical ligation for nanoconjugate performance. Crucially, these findings also extend to other receptor-ligand pairings including EGFR-cetuximab (Fig. S21†), highlighting the versatility of this novel approach and how it may be tailored for diverse applications. This work offers a significant contribution to the ongoing endeavor to refine nanoconjugate design, with the aim of generating more controlled, homogeneous systems that can be readily translated.

## Conflicts of interest

There are no conflicts to declare.

## Supplementary Material

Supplementary information
