# Supplementary material for: Forming next-generation antibody–nanoparticle conjugates through the oriented installation of non-engineered antibody fragments
Source: Chem Sci. 2017 Aug 14;9(1):79–87. doi: 10.1039/c7sc02747h (PMC5869316; doi:10.1039/c7sc02747h)
Supplement: Supplementary file 1 [file SC-009-C7SC02747H-s001.pdf]

Electronic Supplementary Material (ESI) for Chemical Science.
This journal is © The Royal Society of Chemistry 2017

Generating Next-Generation Antibody-Nanoparticle Conjugates through the Oriented Installation of Non-Engineered Antibody Fragments

Michelle K. Greene[1,ǂ], Daniel A. Richards[2,ǂ], João C. F. Nogueira[2], Katrina Campbell[3], Peter Smyth[1], Marcos Fernández[2], Christopher J. Scott[1]*, Vijay Chudasama[2,4]*

[1] *Centre for Cancer Research and Cell Biology, School of Medicine, Dentistry and Biomedical Sciences, Queen's University Belfast, Belfast, UK;* [2] *Department of Chemistry, University College London, London, UK;* [3] *Institute for Global Food Security, School of Biological Sciences, Queen's University Belfast, Belfast, UK,* [4] *Research Institute for Medicines (iMed.ULisboa), Faculty of Pharmacy, Universidade de Lisboa, Lisbon, Portugal*

[ǂ] *These authors contributed equally to this article*

* *E-mails: c.scott@qub.ac.uk; v.chudasama@ucl.ac.uk*

Synthesis General Experimental

All reagents and starting materials were obtained from chemical suppliers, unless specifically stated otherwise, and were used as received. Reactions were monitored by thin layer chromatography using pre-coated SIL G/UV 254 plates purchased from VWR. Flash chromatography was carried out manually using Kieselgel 60 M 0.04/0.063 mm silica gel or automatically using a BioTage Isolera with KP-Snap or KP-Sil columns. NMR spectra were recorded using a Bruker AC300, AC500 or AC600 spectrometer (300 MHz, 500 MHz and 600 MHz respectively). Chemical shifts (δ) are given in ppm units relative to the solvent reference and coupling constants (*J*) are measured in Hertz. Proton (^{1}H) NMR multiplicities are shown as s (singlet), d (doublet), t (triplet), q (quartet), m (multiplet), dd (double doublet), dt (double triplet), etc. HMBC, HSQC and DEPT were employed to aid with accurate assignments. Infrared spectra were recorded on a Perkin Elmer Spectrum 100 FTIR spectrometer (ATR mode). High and low resolution mass spectrometry of organic molecules was provided by the EPSRC Mass Spectrometry facility at Swansea using an LTQ Orbitrap XL.

Chemical Biology General Experimental

All buffers were passed through a microfilter before use to remove particulates and the pH adjusted using 1 M HCl or 1 M NaOH. pH was measured using a Hanna Instruments pH 210 electronic pH meter. When spin filtration devices are mentioned VivaSpin (GE Healthcare) 10,000 Da weight cut off devices were employed. For small scale centrifugation Eppendorf 5415 R and VWR Galaxy 14D microcentrifuges were employed. An Eppendorf Thermomixer Comfort heating block was used for temperature and agitation controlled experiments.

UV-Vis spectroscopy

Protein concentrations were determined photometrically using a ThermoScientific Nanodrop 2000C, and UV-Vis spectra were obtained using a Varian Cary 100 Bio UV-Visible spectrophotometer operating at 21 °C.

SDS-PAGE gels

Non-reducing 12% acrylamide gels were made using standard procedures. A 4% stacking gel was utilised. Samples (15 µM) were mixed 5:1 with a 5×R-250 Dye SDS-loading buffer, heated for 3 minutes at 75 °C and loaded onto the gel with a total volume of 5 µL. Samples were run at constant current (30 mA) for 40 minutes in SDS running buffer and stained with Coomasie.

Protein LCMS

All proteins were prepared for analysis by repeated diafiltration into ammonium acetate buffer (50 mM ammonium acetate, pH 6.9) using VivaSpin sample concentrators (GE Healthcare, 10,000 MWCO) to a concentration of 2 µM. Samples were submitted to the UCL Chemistry Mass Spectrometry Facility at the Chemistry Department, UCL for analysis on the Agilent 6510 QTOF LC-MS system (Agilent, UK). 10 µL of each sample was injected onto a PLRP-S, 1000A, 8 mM, 150 mm × 2.1 mm column, which was maintained at 60 °C. Flow rate was set at 0.600 mL/min. Solvent A is H_2O (0.1% formic acid), solvent B is MeCN (0.1% formic acid), and separation was achieved using a gradient elution. The column effluent was continuously electrosprayed into the capillary ESI source of the Agilent 6510 QTOF mass spectrometer and ESI mass spectra were acquired in positive electrospray ionisation (ESI) mode using the m/z range 1000 – 8000 in profile mode. The raw data was converted to zero charge mass spectra using maximum entropy deconvolution algorithms using MassHunter software (version B.07.00).

Nanoparticle formulation

Polymer blends consisting of 5 mg PLGA-PEG-NHS (Akina AI064; MW ~ 17000:3000 Da) plus 15 mg PLGA RG502H (Evonik Industries), or 5 mg PLGA-PEG-azide (Akina AI085; MW ~ 15000:5000 Da) plus 15 mg PLGA RG502H, were initially dissolved in 1 mL of dichloromethane (DCM). Nanoparticles were fluorescently labelled where required *via* addition of 100 uL of 2 mg/mL rhodamine 6G or 70 uL of 2 mg/mL nile red, both dissolved in DCM, to the organic phase. The organic phase was then injected into 7 mL of 2.5% w/v polyvinyl alcohol in 50 mM 2-(*N*-morpholino) ethanesulfonic acid (MES) hydrate buffer at pH 5, whilst stirring on ice. The emulsion was sonicated in pulse mode for 90 s on ice at an amplitude of 50% using a Model 120 sonic dismembrator (Fisher Scientific) and left stirring overnight to allow organic solvent evaporation. Nanoparticles were centrifuged at 17000 *x g* for 20 min at 4 °C and resuspended in 50 mM MES hydrate buffer at pH 5 *via* sonication. Following a further two centrifugation-resuspension cycles, nanoparticles were adjusted to 1 mg polymer/mL in 50 mM MES hydrate buffer at pH 5 prior to further manipulation.

Nanoparticle functionalisation

Equimolar amounts of native or modified F(ab) were added to NHS- or azide-functionalised nanoparticles resuspended at 1 mg polymer/mL in 50 mM MES hydrate buffer at pH 5, respectively (1050 pmoles of F(ab) per mg polymer, unless otherwise indicated). After stirring at low speed for 2 h, nanoparticles were centrifuged at 12000 *x g* for 20 min at 4 °C and resuspended in PBS *via* sonication. This centrifugation-resuspension cycle was repeated to ensure removal of unbound F(ab) and nanoparticles were adjusted to the required concentration in PBS or water prior to downstream studies.

Nanoparticle characterisation

Nanoparticle size, zeta potential and polydispersity index (PDI) were analysed using a Zetasizer Nano ZS (Malvern Instruments). All measurements were performed following resuspension of nanoparticles at 1 mg polymer/mL in PBS. The bicinchoninic (BCA) protein assay (Thermo Fisher Scientific) was used to quantify F(ab) attachment to nanoparticles, in accordance with the manufacturer's instructions. F(ab) content was determined by reference to standard curves, which were prepared by spiking known amounts of native or modified F(ab) into nude NHS NP or nude azide NP suspensions, respectively. F(ab) conjugation efficiency was calculated as follows: (amount of F(ab) on nanoparticles / amount of F(ab) initially added to nanoparticles)*100. In preparation for environmental scanning electron microscope (ESEM) studies, nanoparticles were resuspended at 5 mg polymer/mL in water and 4 μL droplets were dried overnight on double-sided copper tape fixed to aluminium stubs. Nanoparticles were then sputter-coated with gold and imaged using a Quanta 250 FEG ESEM (FEI) at 30,000× magnification.

Surface plasmon resonance (SPR)

SPR studies were performed on a Biacore Q instrument in HBS-EP running buffer (GE Healthcare) at 25 °C. Recombinant human HER2 or EGFR Fc chimera protein (Sino Biological) was immobilised on a CM5 sensor chip (GE Healthcare), comprised of a surface matrix of carboxymethylated dextran that facilitated ligand coupling *via* carbodiimide chemistry. During immobilisation, carboxyl groups on the sensor chip surface were initially activated by injection of 0.4 M EDC and 0.1 M NHS. Ligand was then injected at 20 μg/mL in 10 mM sodium acetate buffer at pH 4.5, followed by quenching of residual NHS esters with 1 M ethanolamine hydrochloride at pH 8.5. All of the above solutions were injected for a total contact time of 7 min each at a flow rate of 10 μL/min, with typical immobilisation levels of approximately 12,000 resonance units (RU) per flow channel. Nanoparticles were resuspended at 10 mg polymer/mL in HBS-EP running buffer and injected over immobilised ligand for 15 s at a flow rate of 20 μL/min. HER2- and EGFR-coated chip surfaces were regenerated between samples with 50 mM or

25 mM sodium hydroxide, respectively, for 15 s at a flow rate of 20 μL/min. For SPR data presented in tabular or bar chart format, binding responses were calculated relative to baseline, by measuring the change in RU between two report points at 10 s before and 30 s following each injection. Representative sensorgrams show binding responses in absolute RU.

Modified enzyme-linked immunosorbent assay (modified ELISA)
High-binding black 96-well microplates (Greiner Bio-One) were coated with 0.5 μg/mL HER2 Fc chimera protein (Sino Biological) in PBS (100 μL per well) and incubated at 4 °C overnight. Excess protein was discarded and the wells were washed (3×) by immersion in 0.1% v/v Tween 20 in PBS (PBST). Non-specific binding sites were blocked with 1% w/v bovine serum albumin (BSA) in PBS (blocking buffer; 150 μL per well) for 1 h at room temperature, after which washing was repeated as before. Fluorescent rhodamine 6G-loaded nanoparticles ± TRAZ full antibody in blocking buffer (100 μL per well; concentrations stated in figure legends) were then added for 2 h at room temperature. Alternatively, microplates were pre-incubated with 20 μg/mL TRAZ full antibody in blocking buffer (100 μL per well) for 2 h at room temperature, followed by washing (3×) and nanoparticle addition for a further 2 h. Finally, wells were washed (8×), bound nanoparticles were dissolved in a 1:1 volume ratio of ACN:DMSO (50 μL per well) and fluorescence was measured at 530_{ex} / 590_{em} using a Synergy HT plate reader (BioTek).

Confocal microscopy
HCC1954 breast cancer cells were seeded at 30,000 per well on an eight-well glass culture slide (BD Falcon) and allowed to adhere overnight. Cells were treated with 400 μg polymer/mL nile red-loaded nanoparticles ± 200 μg/mL TRAZ full antibody for 18 h, washed in PBS (3×) and fixed with 4% w/v paraformaldehyde in PBS for 20 min. Following further washes in PBS (3×), cells were permeabilised with 0.5% v/v Triton X-100 in PBS for 5 min. Cells were then washed in PBS (3×) and coverslips were positioned following application of Vectashield antifade mounting medium with DAPI (Vector Laboratories). Slides were viewed on a SP5 confocal microscope (Leica Microsystems) equipped with LAS AF software. Images were captured with a ×63 lens zoomed ×1-4, 1024 × 1024 frame and 100 Hz scanning speed.

CellTiter-Glo assay
BT474 breast cancer cells were seeded at 4000 per well on a white 96-well plate and allowed to adhere overnight. Cells were treated with 500 μg polymer/mL nude NHS NP, nude azide NP, native TRAZ F(ab) NP and modified TRAZ F(ab) NP [disulfide]. Treatments also included native TRAZ F(ab) **4** and modified TRAZ F(ab) [disulfide] **5** in free format, which were added at equimolar concentrations to the corresponding nanoformulations. The CellTiter-Glo assay (Promega) was performed at 48, 96 and 144 h following treatment, in accordance with the manufacturer's instructions. At each timepoint, % viability was calculated relative to untreated cells.

Flow cytometry
Cells were washed in PBS (2×) and detached from culture plasticware by addition of 1 mM EDTA and 2% v/v FBS in PBS for 10 min at 37 °C. Following centrifugation at 200 *x g* for 5 min at 4 °C, cells were resuspended in 5% v/v FBS in PBS and incubated with 0.5 µg of anti-human HER2-FITC or anti-mouse IgG1-FITC antibodies (eBioscience) for 30 min at 4 °C. Cells were then washed (3×) in 5% v/v FBS in PBS and HER2 expression was analysed using a FACSCalibur flow cytometer (BD) and FlowJo software.

Statistical analysis

Statistical significance was determined by one-way analysis of variance (ANOVA) with Tukey's post-hoc test, using GraphPad Prism software (version 6.0c). Significant differences are denoted by asterisks on the relevant graphs and defined in the corresponding figure legends.

***tert*-Butyl 1-methylhydrazine-1-carboxylate**[1]

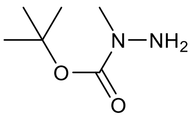

To a stirring solution of *N*-methylhydrazine (1.73 g, 37.5 mmol) in CH_2Cl_2 (100 mL) at 0 °C was added dropwise di-*tert*-butyl dicarbonate (8.12 g, 37.5 mmol, pre-dissolved in CH_2Cl_2 (40 mL)) over 40 min. The mixture was allowed to warm to room temperature (21 °C) and stirred for a further 3 h. After this time, the reaction mixture was concentrated *in vacuo* to yield *tert*-butyl-1-methylhydrazine-1-carboxylate as a yellow liquid without further purification (5.15 g, 35.3 mmol, 94%). ^{1}H NMR ($CDCl_3$, 600 MHz) δ 4.01 (br s, 2H), 3.02 (s, 3H), 1.44 (s, 9H); ^{13}C NMR ($CDCl_3$, 150 MHz) δ 157.2 (C), 80.4 (C), 38.3 (CH_3), 28.6 (CH_3); IR (thin film) 3330, 3220, 2977, 2932, 1668 cm^{-1}.

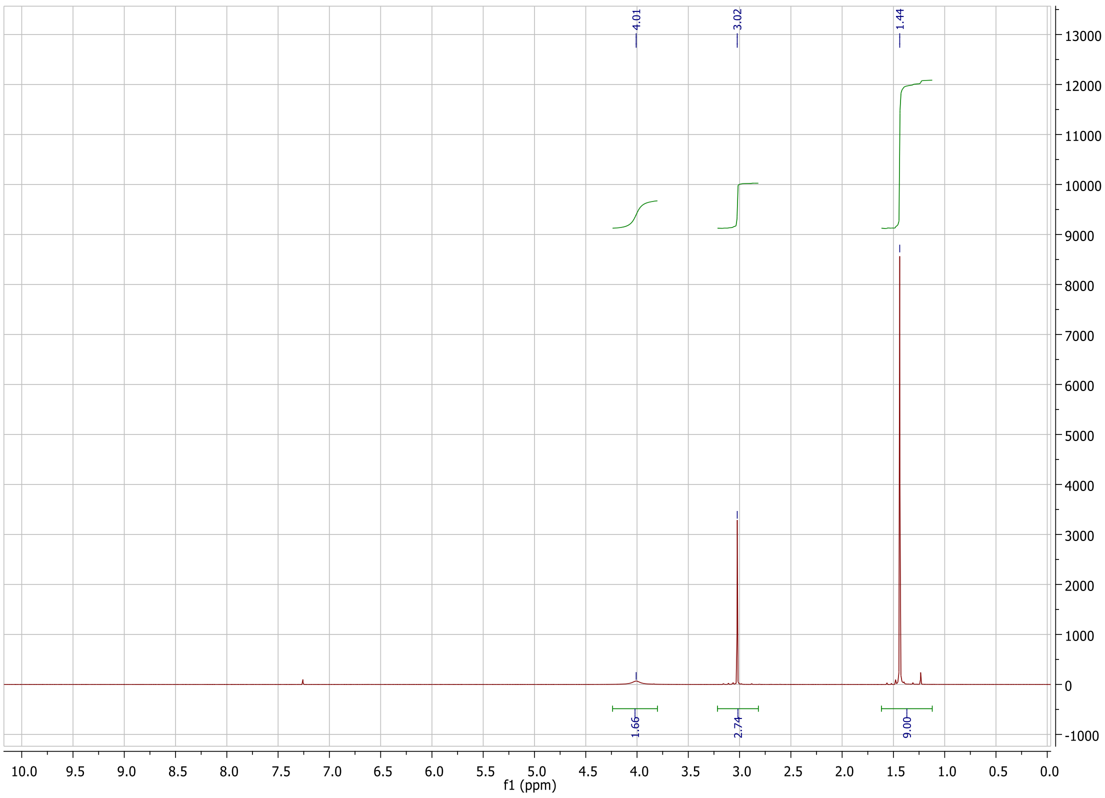

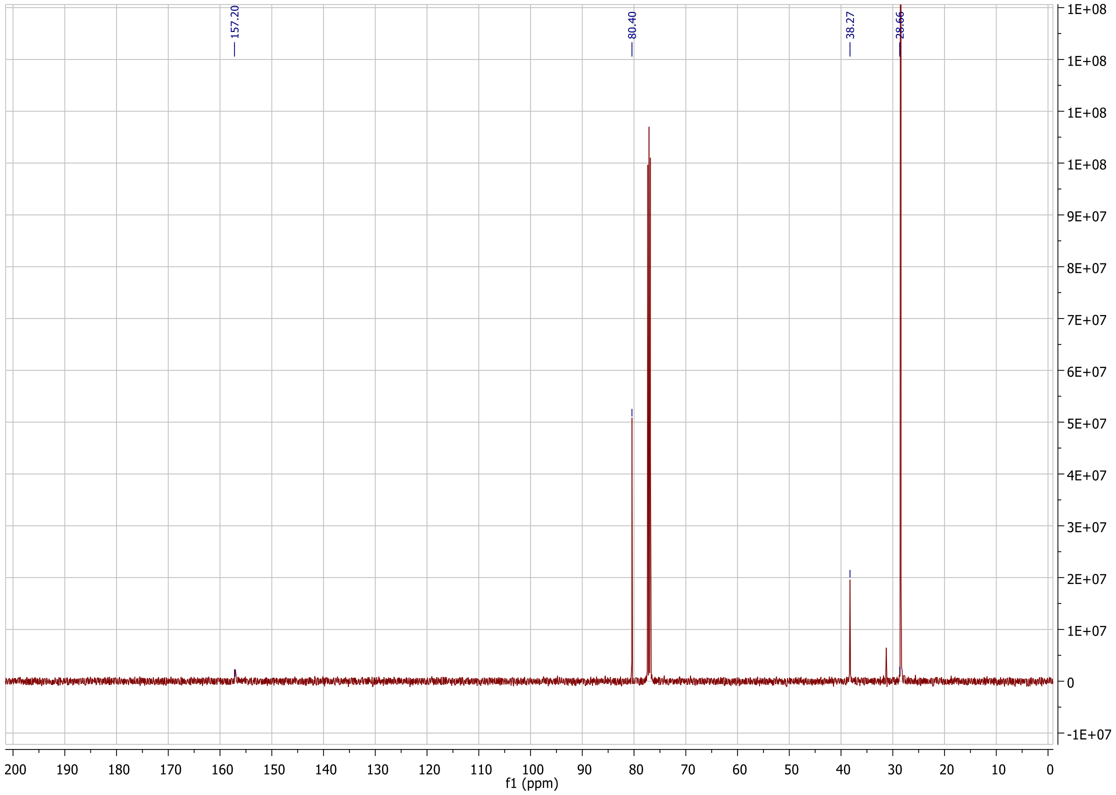

Figure S1: ^{1}H and ^{13}C NMR data for *tert*-butyl 1-methylhydrazine-1-carboxylate.

((*tert*-Butoxycarbonyl)(methyl)amino)glycine 1[2]

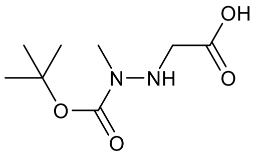

To a stirring solution of *tert*-butyl 1-methylhydrazine-1-carboxylate (585 mg, 4.00 mmol) in *i*-PrOH (10 mL) was added glyoxylic acid (296 mg, 4.00 mmol), and the reaction mixture stirred at 21 °C for 5.5 h. 10% Pd/C (80 mg) was subsequently added and the flask placed under vacuum to remove all air. The flask was filled with an atmosphere of H_2 *via* a balloon and the suspension stirred for 24 h. After this time, the solution was filtered through Celite® 545 and the reaction mixture concentrated *in vacuo* to yield a crude oil. Trituration with CH_2Cl_2 afforded ((*tert*-butoxycarbonyl)(methyl)amino)glycine **1** as a yellow foam (170 mg, 0.80 mmol, 20%). ^{1}H NMR (DMSO-d_6, 400 MHz) δ 3.46 (s, 2H), 2.93 (s, 3H), 1.40 (s, 9H); ^{13}C NMR (DMSO-d_6, 100 MHz) δ 171.9 (C), 79.5 (C), 50.9 (CH_2), 28.3 (CH_3), 28.0 (CH_3); IR (thin film) 3299, 2977, 2931, 1668 cm^{-1}.

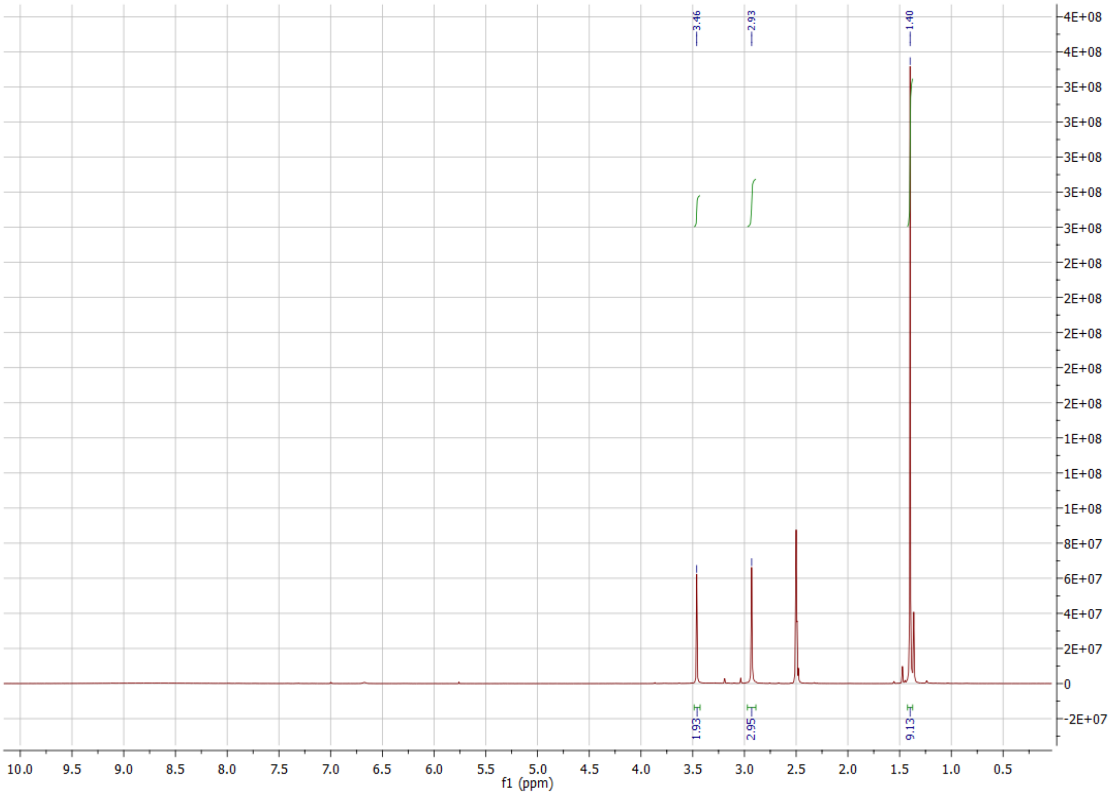

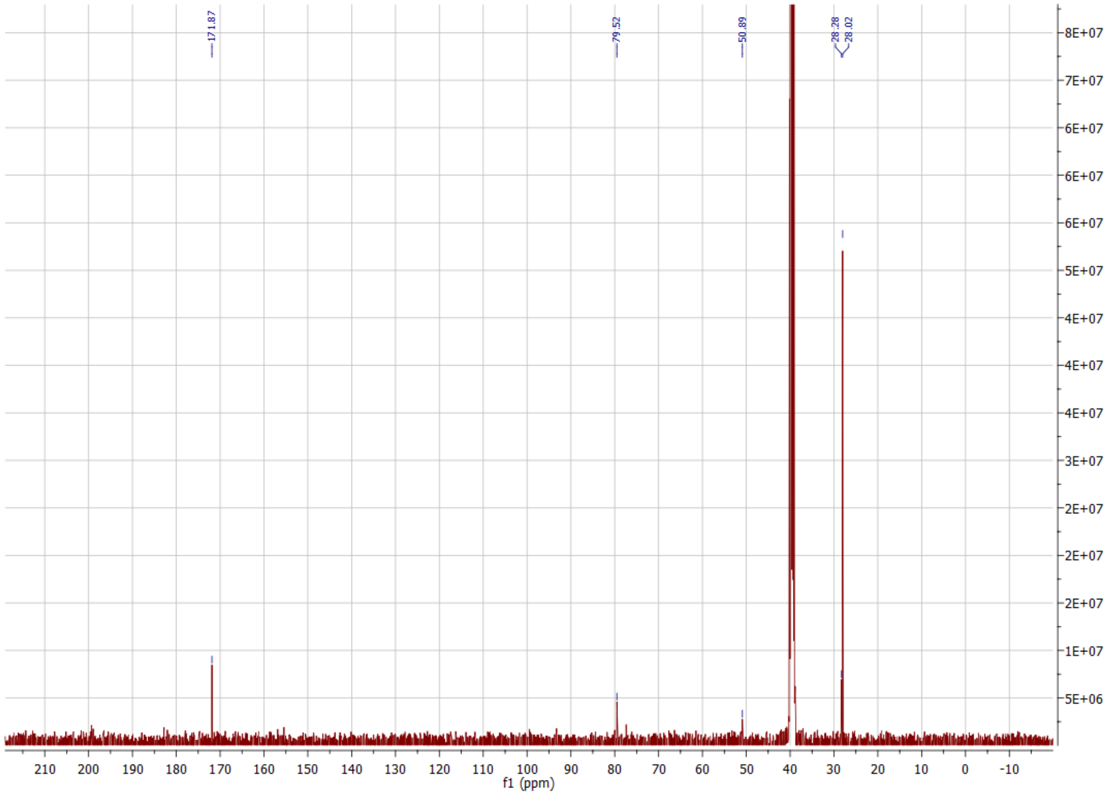

Figure S2: ^{1}H and ^{13}C NMR data for ((*tert*-butoxycarbonyl)(methyl)amino)glycine **1**.

2-(4,5-Dibromo-2-methyl-3,6-dioxo-3,6-dihydropyridazin-1(2*H*)-yl)acetic acid 2[3]

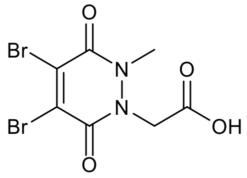

To a stirring solution of *tert*-butyl 1-methylhydrazine-1-carboxylate **1** (245 mg, 1.2 mmol) in AcOH (10 mL) was added dibromomaleic acid (0.55 g, 2.0 mmol), and the reaction mixture was heated under reflux for 24 h. All solvent was removed *in vacuo* with toluene co-evaporation, the crude material dissolved in EtOAc (50 mL), washed with H_2O (3 × 20 mL), and dried ($MgSO_4$). Purification by flash column chromatography (3% MeOH/CH_2Cl_2 with 1% AcOH) yielded 2-(4,5-dibromo-2-methyl-3,6-dioxo-3,6-dihydropyridazin-1(2*H*)-yl)acetic acid **2** (252 mg ,0.74 mmol, 62%) as a white solid: m.p. 204.5-206.5 °C (*lit m.p.* 210-214 °C); ^{1}H NMR (600 MHz, MeOD) δ 4.96 (s, 2H), 3.62 (s, 3H); ^{13}C NMR (150 MHz, MeOD) δ 170.2 (C), 154.8 (C), 154.0 (C), 137.4 (C), 135.7 (C), 49.5 (CH_2), 35.0 (CH_3); IR (solid) 3023, 2969, 1731, 1662 cm^{-1}; LRMS (ES^-) 342 (50, $[M^{81}Br^{81}Br{-}H]^-$), 340 (100, $[M^{81}Br^{79}Br{-}H]^-$), 338 (50, $[M^{79}Br^{79}Br{-}H]^-$); HRMS (ES^-) calcd for $C_7H_5N_2O_4{}^{79}Br_2$ $[M^{79}Br^{79}Br{-}H]^-$ 337.8538, observed 337.8540.

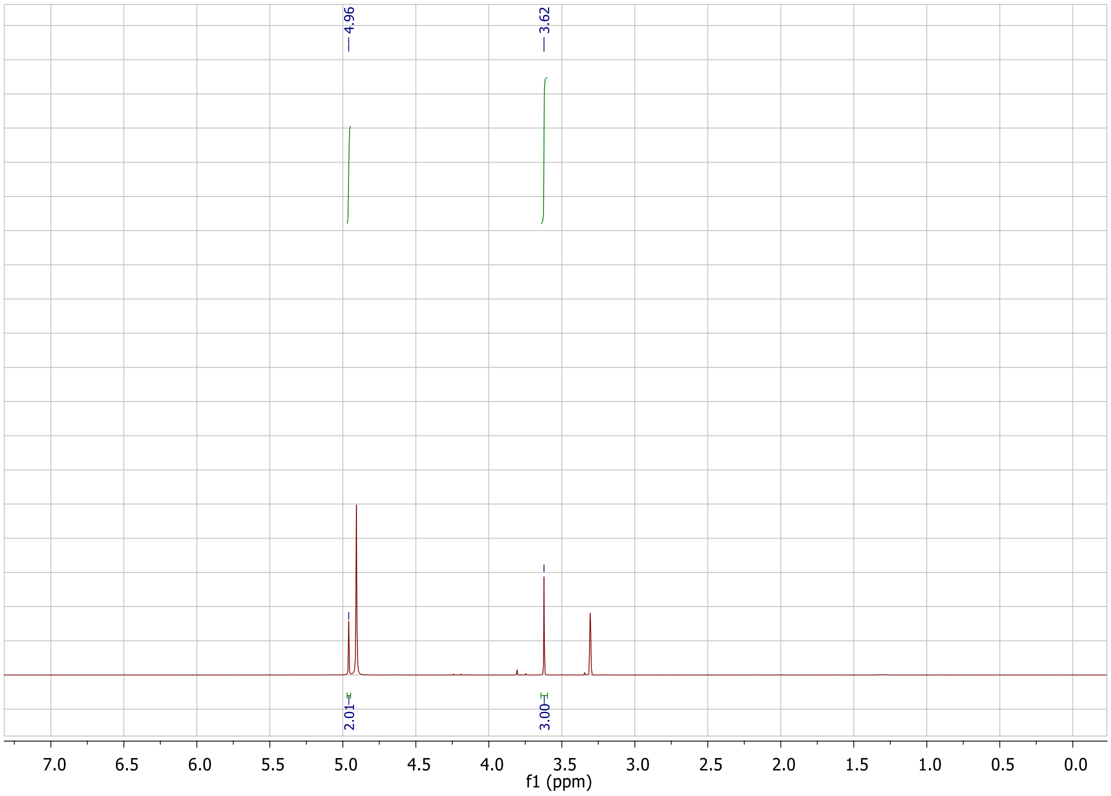

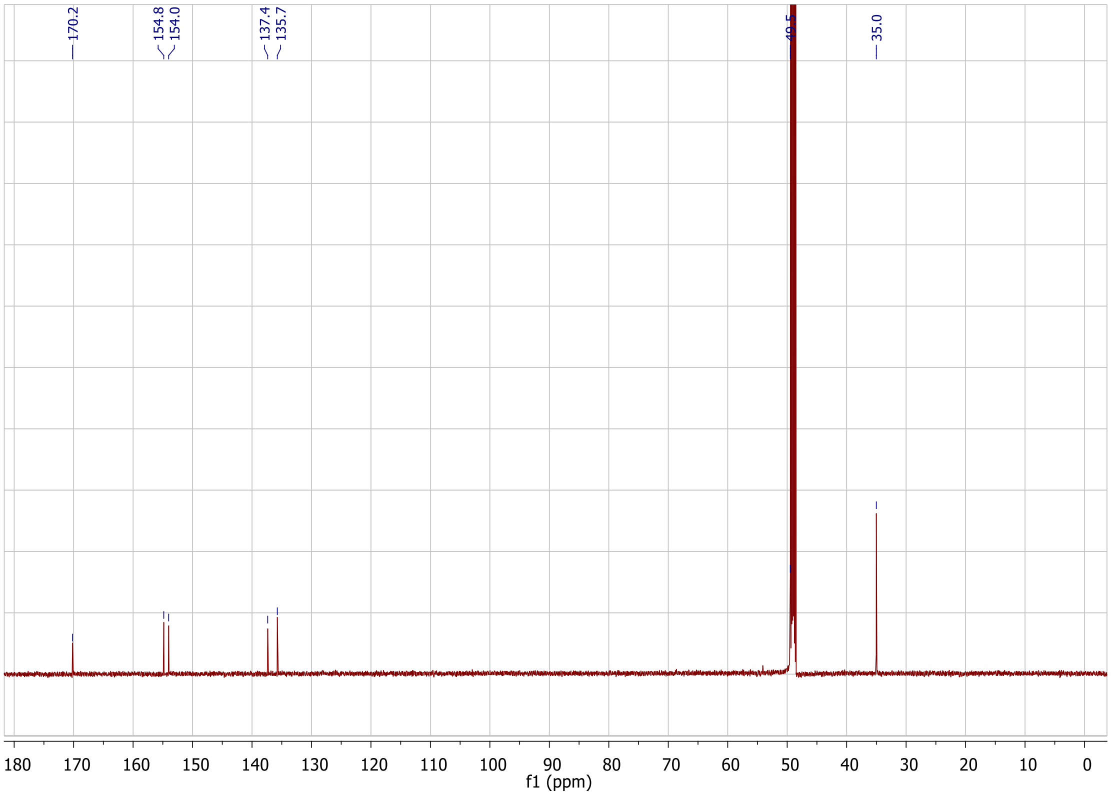

Figure S3: ^{1}H and ^{13}C NMR data for 2-(4,5-dibromo-2-methyl-3,6-dihydropyridazin-1(2*H*)-yl)acetic acid **2**.

Bicyclo[6.1.0]non-4-yn-9-ylmethyl (2-(2-(2-(2-(4,5-dibromo-2-methyl-3,6-dioxo-3,6-dihydropyridazin-1(2*H*)-yl)acetamido)ethoxy)ethoxy)ethyl)carbamate 3

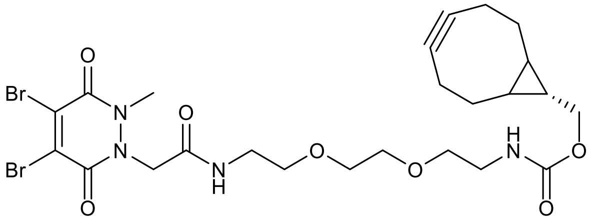

To a solution of 2-(4,5-dibromo-2-methyl-3,6-dioxo-3,6-dihydropyridazin-1(2*H*)-yl)acetic acid **2** (43 mg, 0.125 mmol), PyBOP (70 mg, 0.14 mmol), and DIPEA (49 μL, 0.28 mmol) in CH_2Cl_2 (2.5 mL), was added *N*-[(1R,8S,9s)-bicyclo[6.1.0]non-4-yn-9-ylmethyloxycarbonyl]-1,8-diamino-3,6-dioxaoctane (40 mg, 0.125 mmol). The resulting solution was stirred at 21 °C for 16 h. Then the reaction mixture was diluted with H_2O (7 mL), extracted with EtOAc (3 × 7 mL), the combined organic layers were dried ($MgSO_4$) and concentrated *in vacuo*. The crude residue was purified by flash column chromatography (0 – 5% MeOH in EtOAc) to afford bicyclo[6.1.0]non-4-yn-9-ylmethyl (2-(2-(2-(2-(4,5-dibromo-2-methyl-3,6-dioxo-3,6-dihydropyridazin-1(2*H*)-yl)acetamido)ethoxy)ethoxy)ethyl)carbamate **3** (32 mg, 0.050 mmol, 40%) as a white solid: ^{1}H NMR (600 MHz, $CDCl_3$) δ 8.34 (br. s, 0.5H), 7.00 (br. s, 0.5H), 5.96 (br. s, 0.5H), 5.29 (br. s, 0.5H), 4.85–4.73 (m, 2H), 4.12 (d, *J* = 8.2 Hz, 2H), 3.76–3.50 (m, 11H), 3.50–3.43 (m, 2H), 3.40–3.30 (m, 2H), 2.31–2.17 (m, 6H), 1. 61–1.51 (m, 2H), 1.41–1.24 (m, 1H), 1.01–0.85 (m, 2H); ^{13}C NMR (150 MHz, $CDCl_3$) (major rotamer) δ 165.6 (C), 157.8 (C), 153.4 (C), 152.5 (C), 137.0 (C), 134.8 (C), 98.9 (C), 77.4 (CH_2), 77.2 (CH_2), 77.0 (CH_2), 70.8 (CH_2), 70.6 (CH_2), 70.5 (CH_2), 70.3 (CH_2), 70.2 (CH_2), 70.0 (CH_2), 69.6 (CH_2), 69.4 (CH_2), 63.0 (CH_2), 50.9 (CH_2), 50.3 (CH_2), 40.9 (CH_2), 39.7 (CH_2), 35.0 (CH_3), 29.1 (CH), 21.5 (CH_2), 20.2 (CH), 17.8 (CH_2); IR (thin film) 3338, 2925, 1685, 1633 cm^{-1}; LRMS (ES^+) 651 (50, $[M^{81}Br^{81}Br+H]^+$), 649 (100, $[M^{81}Br^{79}Br+H]^+$), 647 (50, $[M^{79}Br^{79}Br+H]^+$); HRMS (ES^+) calcd for $C_{24}H_{33}N_4O_7{}^{79}Br_2$ $[M^{79}Br^{79}Br+H]^+$ 647.0716, observed 647.0713.

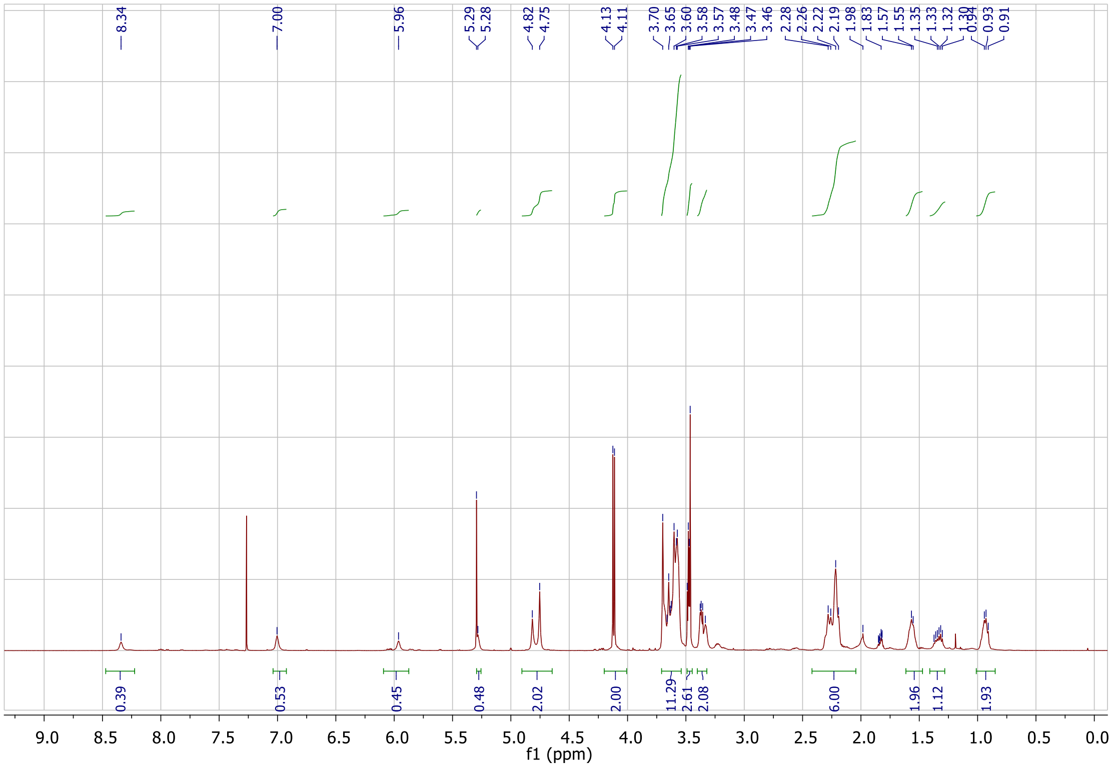

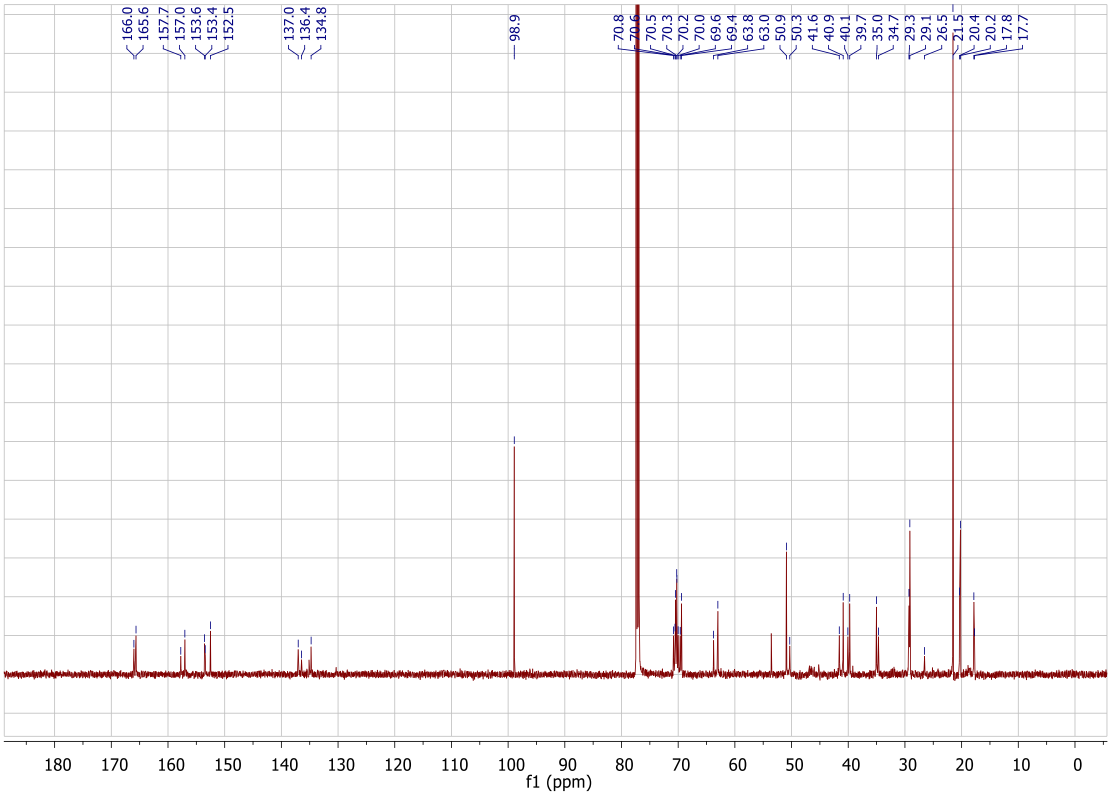

Figure S4: ^{1}H and ^{13}C NMR data for bicyclo[6.1.0]non-4-yn-9-ylmethyl (2-(2-(2-(2-(4,5-dibromo-2-methyl-3,6-dioxo-3,6-dihydropyridazin-1(2*H*)-yl)acetamido)ethoxy)ethoxy)ethyl)carbamate **3**.

2,5-Dioxopyrrolidin-1-yl 1-((1R,8S,9S)-bicyclo[6.1.0]non-4-yn-9-yl)-3,14-dioxo-2,7,10-trioxa-4,13-diazaoctadecan-18-oate

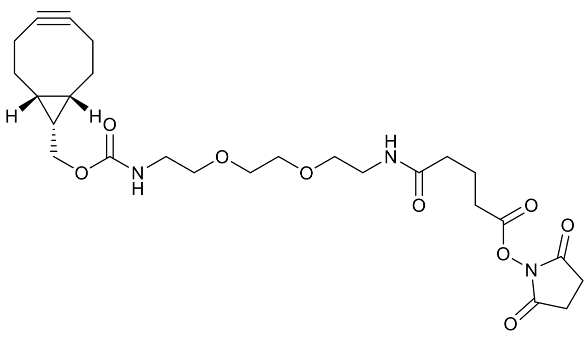

To a stirring solution of ((1R,8S,9S)-bicyclo[6.1.0]non-4-yn-9-yl)methyl (2-(2-(2-aminoethoxy)ethoxy)ethyl)carbamate (100 mg, 0.308 mmol) in CH_2Cl_2 (150 mL), were added glutaric anhydride (38.7 mg, 0.339 mmol) and Et_3N (0.129 mL, 0.925 mmol). The reaction was stirred for 2 h followed by the addition of DSC (119 mg g, 0.462 mmol). After 2 h the reaction was diluted with CH_2Cl_2 (20 mL), washed with H_2O (3 × 30 mL) and dried over $MgSO_4$. The organic layer was filtered, all solvent removed *in vacuo* and the crude material purified by flash column chromatography (0 – 6% MeOH in EtOAc) to afford 2,5-dioxopyrrolidin-1-yl 1-((1R,8S,9S)-bicyclo[6.1.0]non-4-yn-9-yl)-3,14-dioxo-2,7,10-trioxa-4,13-diazaoctadecan-18-oate (70.0 mg, 0.131 mmol, 42%) as a clear gum. ^{1}H NMR (600 MHz, $CDCl_3$) δ 6.30 (s, 1H), 5.24 (s, 1H), 4.14 (d, *J* = 8.1 Hz, 2H), 3.61 (s, 4H), 3.57–3.52 (m, 4H), 3.46 (q, *J* = 5.3 Hz, 2H), 3.35 (q, *J* = 5.0 Hz, 2H). 2.85 (br s, 4H), 2.67 (t, *J* = 6.9 Hz, 2H), 2.34-2.11 (m, 7H), 2.09 (qn, *J* = 7.0 Hz, 2H), 1.59 (m, 2H), 1.34 (t, *J* = 9.0 Hz, 1H), 0.96–0.93 (m, 2H). ^{13}C NMR (150 MHz, $CDCl_3$) δ 171.8 (C), 169.4 (C), 168.5 (C), 156.9 (C), 98.9 (C), 70.3–70.0 (CH_2 × 4) 62.9 (CH_2), 40.9 (CH), 39.3 (CH_2), 34.6 (CH_2), 30.1 (CH_2), 29.2 (CH_2), 25.7 (CH_2), 21.5 (CH_2), 20.9 (CH_2), 20.2 (CH_2), 17.9 (CH); IR (thin film) 3324, 3061, 2911, 2872, 1684, 1633, 1533 cm^{-1}; LRMS (ES^+) 460 (85, $[M\text{-}NHS+Na]^+$), 438 (100, $[M\text{-}NHS]^+$); HRMS (ES^+) calcd for $C_{26}H_{41}N_4O_9$ $[M+NH_4]^+$ 553.2868, observed 553.2861.

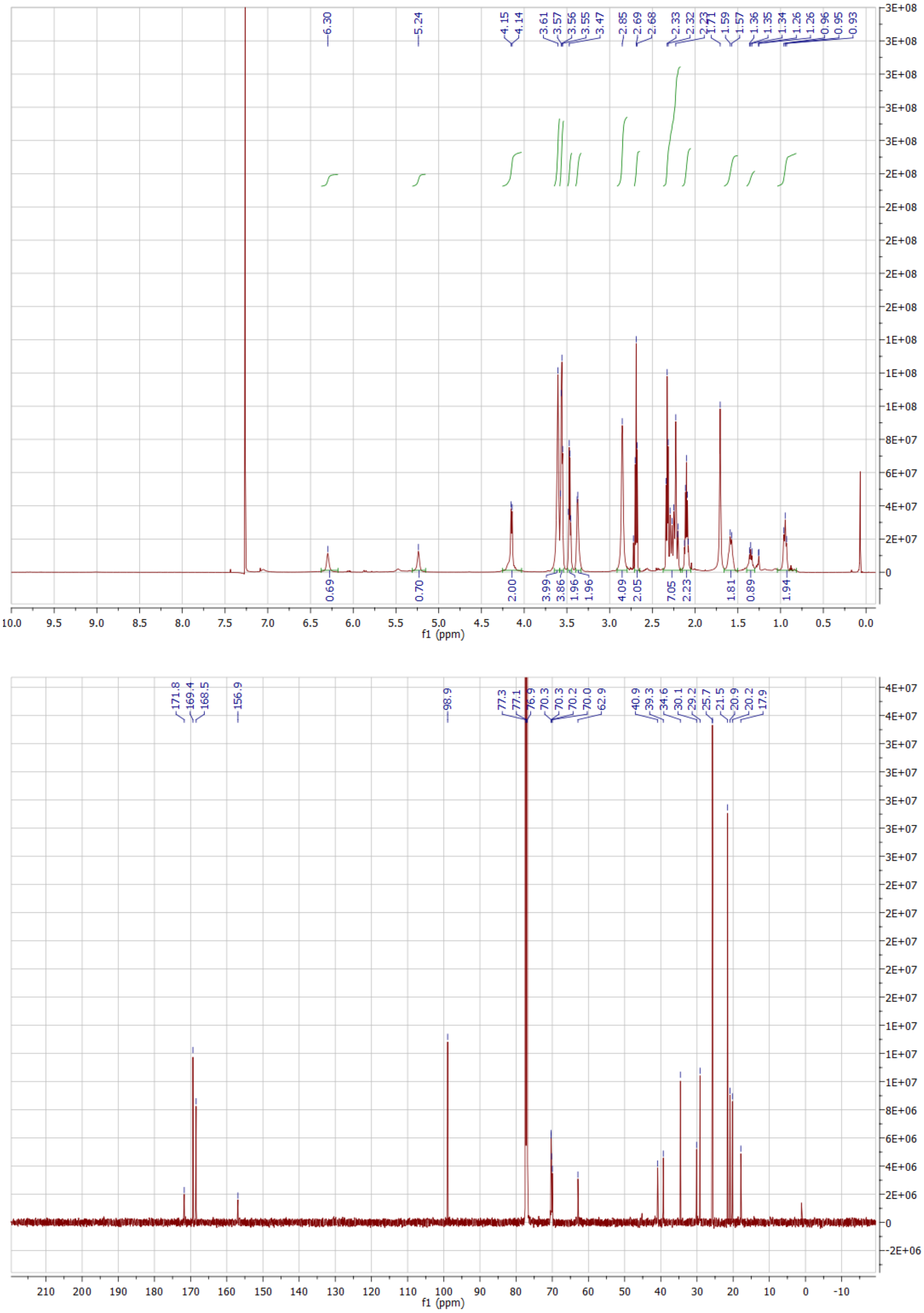

Figure S5: ^{1}H and ^{13}C NMR data for 2,5-dioxopyrrolidin-1-yl 1-((1R,8S,9S)-bicyclo[6.1.0]non-4-yn-9-yl)-3,14-dioxo-2,7,10-trioxa-4,13-diazaoctadecan-18-oate.

TRAZ F(ab) 4

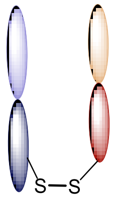

Immobilized pepsin (0.15 mL) was washed with acetate buffer (20 mM sodium acetate trihydrate, pH 3.1) four times and traztuzumab (TRAZ) (0.5 mL, 6.41 mg·mL^{-1}, 44.2 μM, in acetate buffer) was added. The mixture was incubated for 5 h at 37 °C whilst shaking (1100 rpm). The resin was separated from the digest using a filter column, and washed with digest buffer (50 mM phosphate, 150 mM NaCl, 1 mM EDTA, pH = 6.8) three times. The digest was combined with the washes and the combined mixture buffer swapped into digest buffer *via* diafiltration (4 × 15 mL, GE Healthcare, 10,000 MWCO) to remove impurities. This yielded TRAZ F(ab')$_2$ (0.5 mL, 43.6 μM, 99% yield). Concentration was determined photometrically using ε_{280} = 140000 M^{-1}·cm^{-1}. Immobilized papain (0.5 mL, 0.25 mg·mL^{-1}) was activated with 10 mM DTT in digest buffer whilst shaking (1100 rpm) for 1 h at 37 °C. The resin was washed with digest buffer (without DTT) four times and TRAZ F(ab')$_2$ was added (0.5 mL, 43.6 μM). The mixture was incubated for 16 h at 37 °C whilst shaking (1100 rpm). The resin was separated from the digest using a filter column, and washed with BBS (25 mM sodium borate, 25 mM NaCl, 0.5 mM EDTA, pH 8.0) three times. The digest was combined with the washes and the buffer exchanged completely for BBS using diafiltration columns (4 × 15 mL, GE Healthcare, 10,000 MWCO) to remove impurities. This yielded TRAZ F(ab) **4** (0.5 mL, 61 μM, 70% yield) as confirmed by SDS-PAGE and LCMS. Concentration was determined photometrically using ε_{280} = 68590 M^{-1}·cm^{-1}. Observed mass 47637 Da.

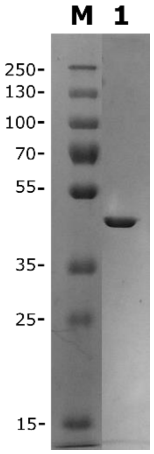

Figure S6: SDS-PAGE gel. M) Molecular weight marker in KDa; 1) TRAZ F(ab) **4**.

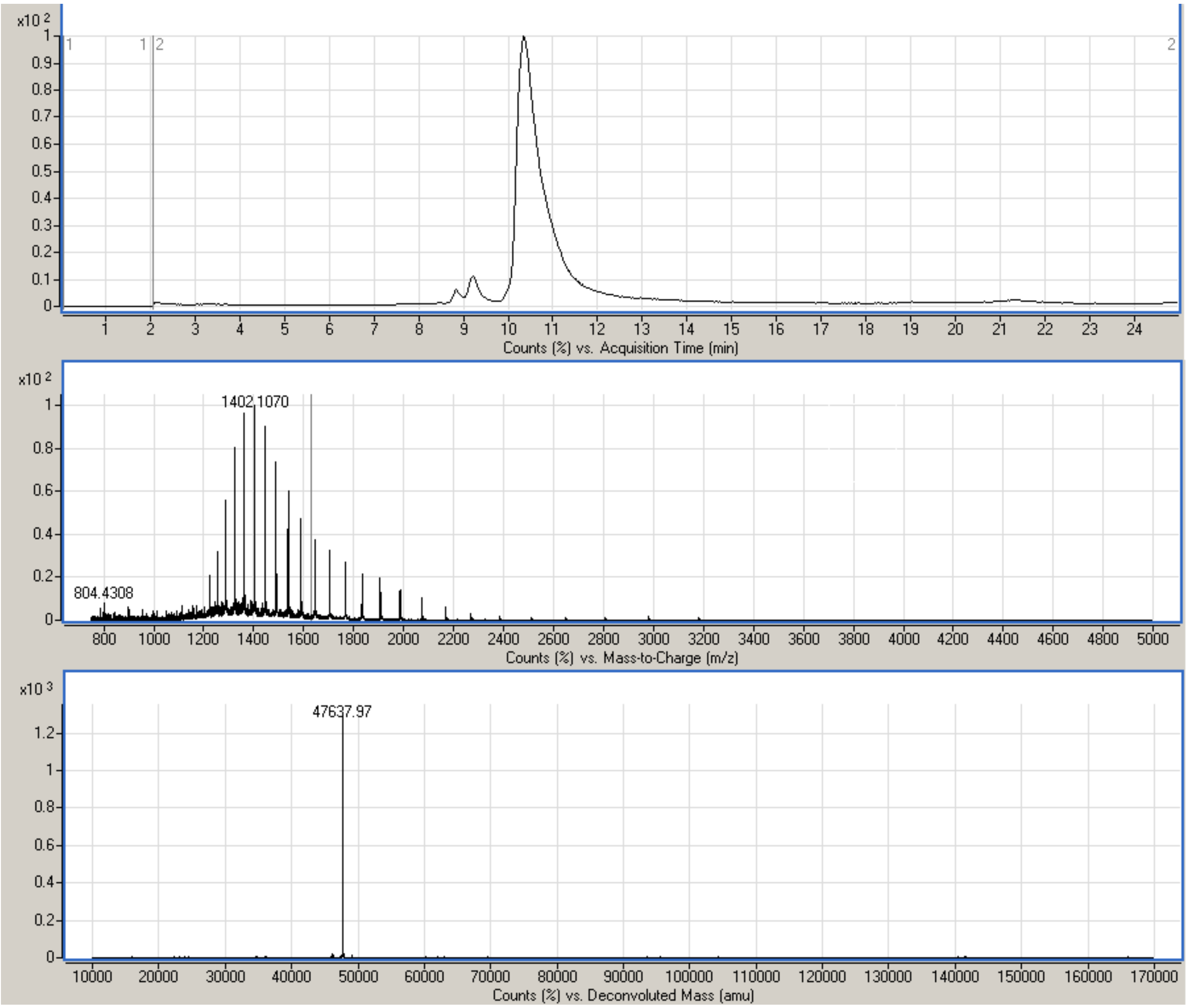

Figure S7: TIC, non-deconvoluted and deconvoluted MS data for TRAZ F(ab) **4**.

TRAZ F(ab) [disulfide] 5

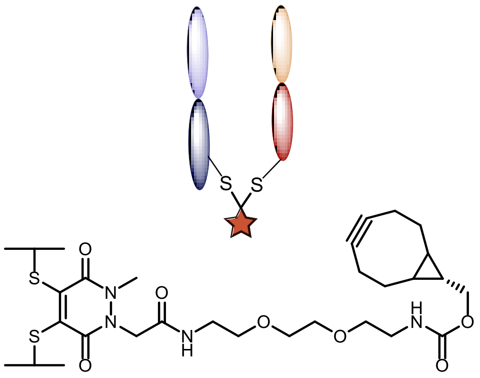

TCEP.HCl (2.5 µL, 20 mM in deionised water, 5 eq.) was added to a solution of TRAZ F(ab) **4** (500 µL, 20 µM) in BBS (25 mM sodium borate, 25 mM NaCl, 0.5 mM EDTA, 2% DMSO, pH 8.0) which had been pre-treated with pyridazinedione **3** (5.0 µL, 20 mM in DMSO, 10 eq.) and stored at 4 °C for 1 h previously. The reaction mixture was then incubated at 4 °C for 15 h. Excess reagents were removed by repeated diafiltration into PBS (pH = 7.4) using VivaSpin sample concentrators (GE Healthcare, 10,000 MWCO). The samples were analysed by SDS-PAGE gel and LCMS. Observed mass 48125 Da, expected mass 48125 Da.

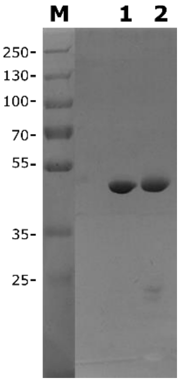

Figure S8: SDS-PAGE gel. M) Molecular weight marker; 1) TRAZ F(ab) **4**; 2) TRAZ F(ab) [disulfide] **5**.

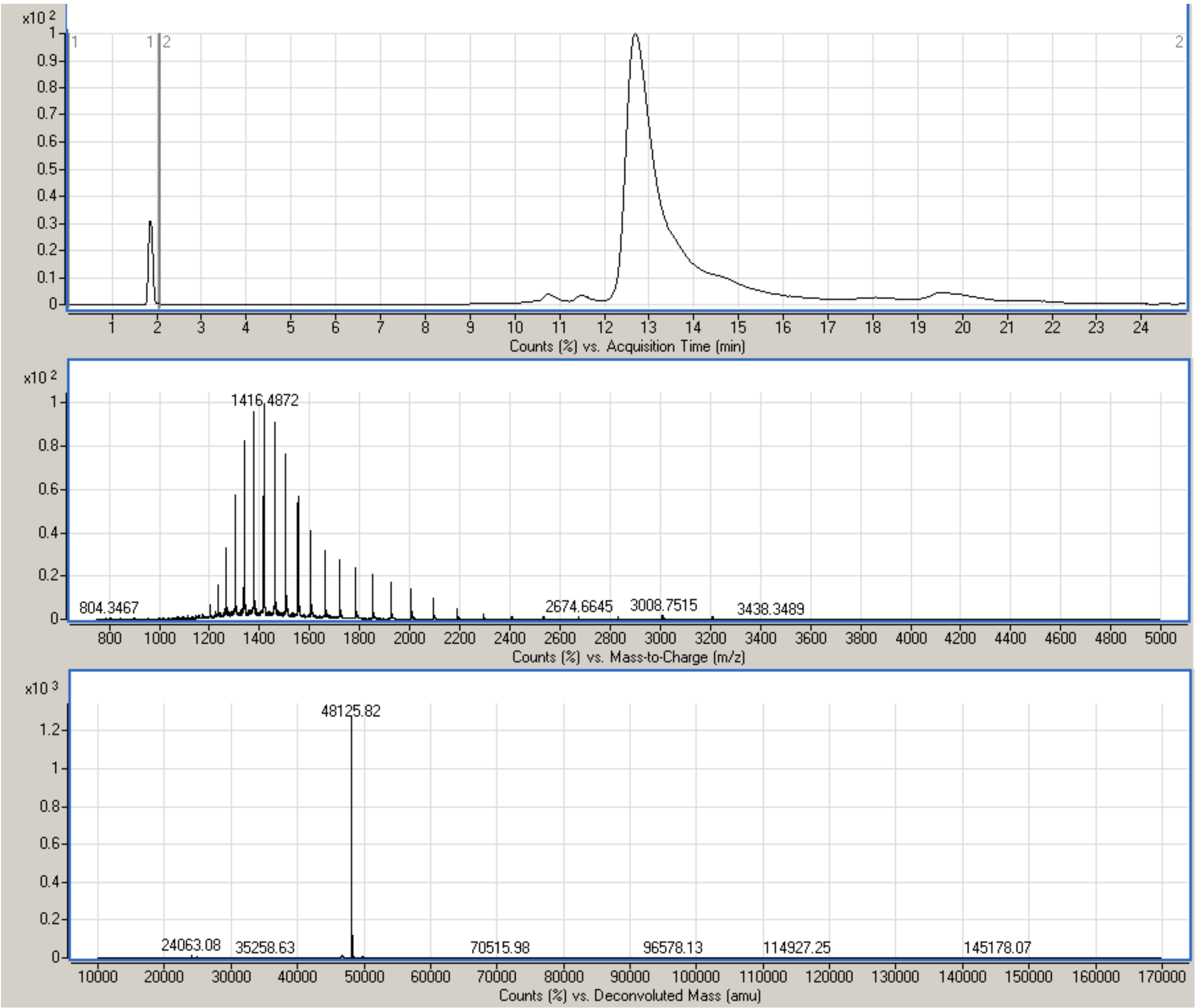

Figure S9: TIC, non-deconvoluted and deconvoluted MS data for TRAZ F(ab) [disulfide] **5**.

TRAZ F(ab) [disulfide]-Alexafluor488® 6

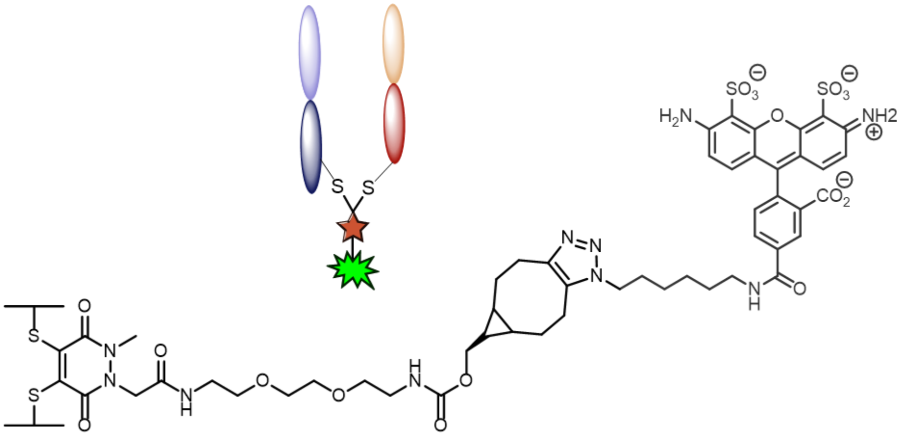

To a solution of TRAZ F(ab) [disulfide] **5** (50 µL, 20 µM, 1 eq.) in BBS (+3% DMSO) was added Alexafluor488®-azide (0.2 µL, 20 mM in DMSO, 4 eq.) and the reaction mixture incubated at 25 °C for 6 h. The excess reagents were then removed by repeated diafiltration into fresh buffer using VivaSpin sample concentrators (GE Healthcare, 10,000 MWCO). Successful click was confirmed by SDS-PAGE, UV-Vis, and LCMS. Observed mass 48,784 Da, expected mass 48,792 Da. UV-vis spectroscopy was used to determine a loading of 1 : 4.8 (TRAZ F(ab) : strained alkyne). An extinction coefficient of ε_{494} = 71000 M^{-1} cm^{-1} was used for Alexafluor488®. A correction factor of 0.11 × A_{494} was used to correct at A_{280} for Alexafluor488®.

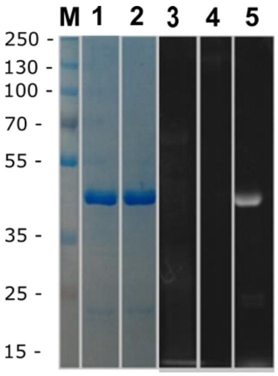

Figure S10: SDS-PAGE gel. M) Molecular weight marker in KDa: 1) TRAZ F(ab) [disulfide] **5**; 2) TRAZ F(ab) [disulfide]-Alexafluor488® **6**; 3) Molecular weight marker in KDa; 4) TRAZ F(ab) [disulfide] **5**; 5) TRAZ F(ab) [disulfide]-Alexafluor488® **6**. Lanes M, 1 and 2 were imaged using a Bio-Rad Gel Doc™, and lanes 3-5 were imaged using a BioDoc-It™ Imaging System.

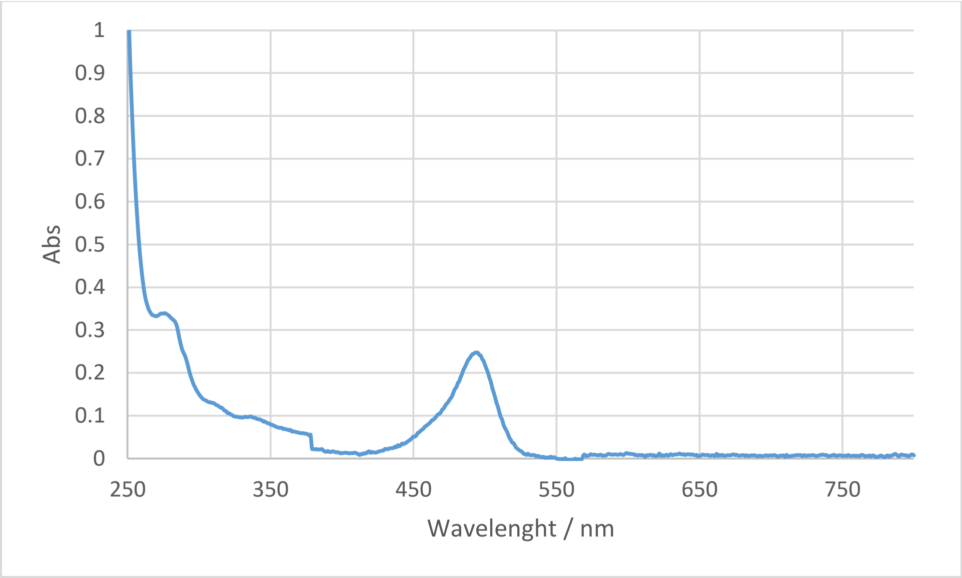

Figure S11: UV-Vis data for TRAZ F(ab) [disulfide]-Alexafluor488® **6**, FAR = 0.9.

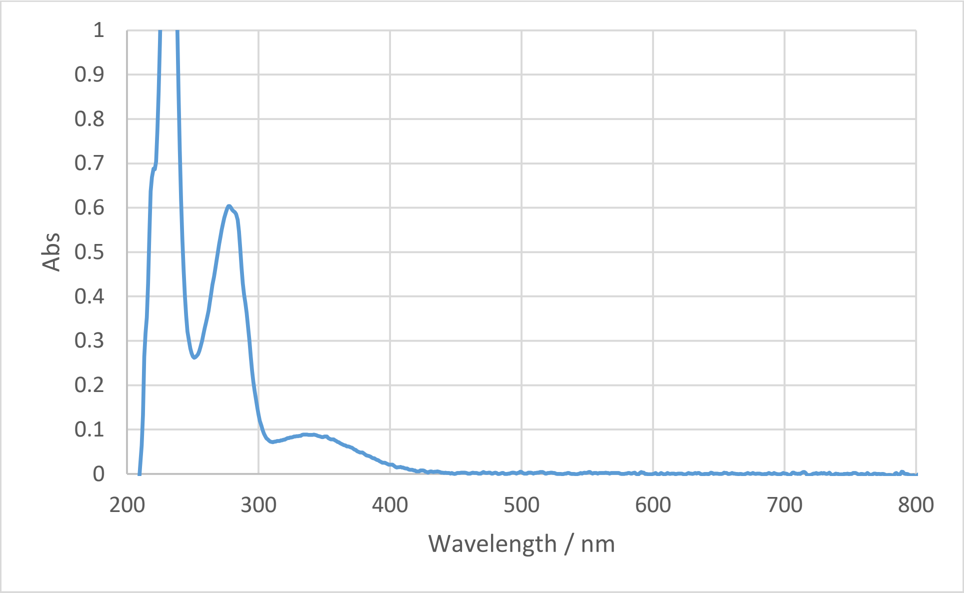

Figure S11a: Control UV-Vis data for TRAZ F(ab) [disulfide] **5** (no Alexafluor488®-azide).

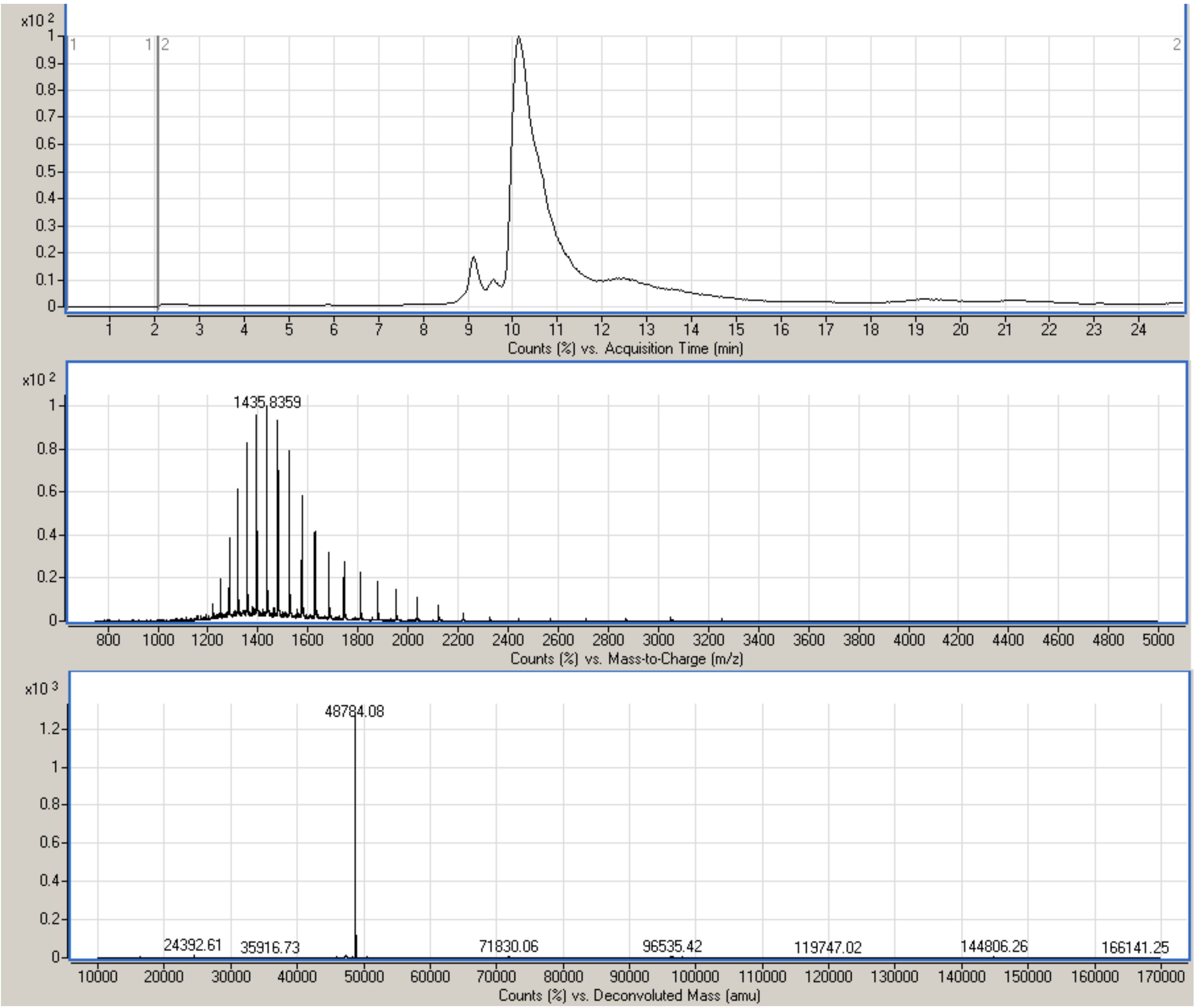

Figure S12: TIC, non-deconvoluted and deconvoluted MS data for TRAZ F(ab) [disulfide]-Alexafluor488 **6**.

TRAZ F(ab) [lys]

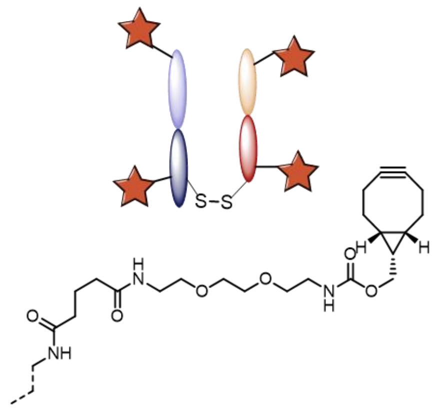

2,5-dioxopyrrolidin-1-yl 1-((1R,8S,9S)-bicyclo[6.1.0]non-4-yn-9-yl)-3,14-dioxo-2,7,10-trioxa-4,13-diazaoctadecan-18-oate (10 µL, 60 mM in DMSO, 40 equiv.) was added to a solution of TRAZ F(ab) **4** (250 µL, 60 µM in BBS pH = 8) and incubated at 37 °C for 4 hours. Excess reagents were removed by repeated diafiltration into PBS (pH = 7.4) using VivaSpin sample concentrators (GE Healthcare, 10,000 MWCO). A small sample (40 µL at 20 µM) was reacted with Alexafluor488®-azide (2 µL, 50 equiv., 20 mM in DMSO) at room temperature for 16 hours to check for the presence of a strained alkyne. Excess reagents were removed by repeated diafiltration into PBS (pH = 7.4) using VivaSpin sample concentrators (GE Healthcare, 10,000 MWCO). The samples were analysed by SDS-PAGE, and UV-vis spectroscopy was used to determine a loading of 1 : 4.8 (TRAZ F(ab) : strained alkyne). An extinction coefficient of $\varepsilon_{494} = 71000\ M^{-1}\ cm^{-1}$ was used for Alexafluor488®. A correction factor of $0.11 \times A_{494}$ was used to correct at A_{280} for Alexafluor488®.

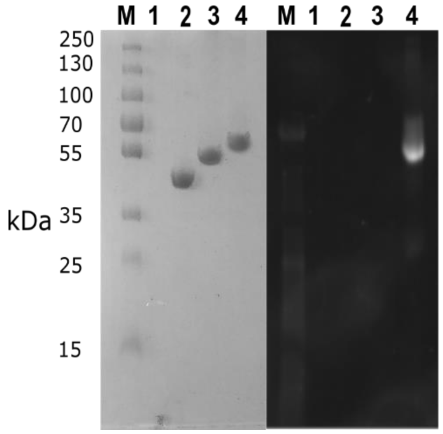

Figure S13: SDS-PAGE gel. M) Molecular weight marker in KDa: 1) Empty; 2) TRAZ F(ab); 3) TRAZ F(ab) [lys] 4) TRAZ F(ab) [lys]-Alexafluor488®. LHS lanes M, 1-4 were imaged using Bio-Rad Gel Doc™, and RHS lanes M, 1-4 were imaged using a BioDoc-It™ Imaging System.

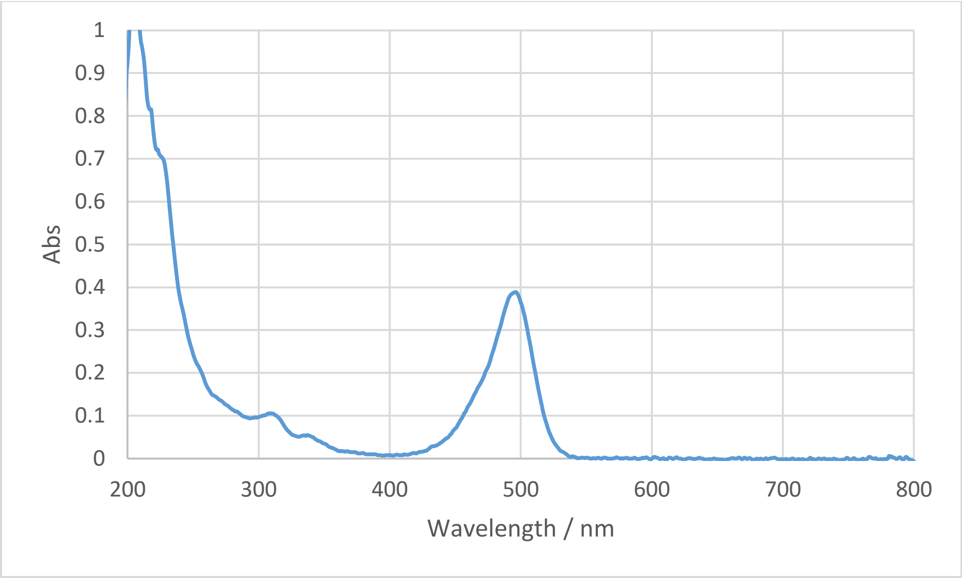

Figure S14: UV-Vis data for TRAZ F(ab) [lys]-Alexafluor488®.

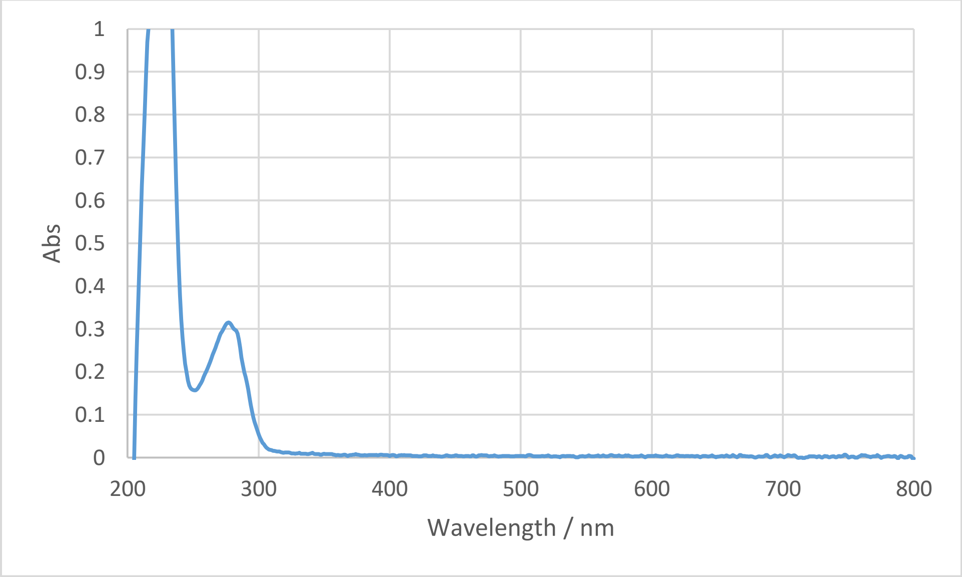

Figure S14a: UV-Vis data for TRAZ F(ab) [lys] (no Alexafluor488®-azide).

CTX F(ab)

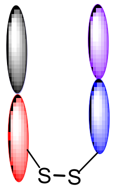

Immobilized pepsin (0.15 mL) was washed with acetate buffer (20 mM sodium acetate trihydrate, pH 3.1) four times and cetuximab (CTX) (0.5 mL, 2.00 mg·mL^{-1}, 13.8 μM, in acetate buffer) was added. The mixture was incubated for 5 h at 37 °C whilst shaking (1100 rpm). The resin was separated from the digest using a filter column, and washed with digest buffer (50 mM phosphate, 150 mM NaCl, 1 mM EDTA, pH = 6.8) three times. The digest was combined with the washes and the combined mixture buffer swapped into digest buffer (25 mM sodium borate, 25 mM NaCl, pH 8.0) via diafiltration (4 × 15 mL, GE Healthcare, 10,000 MWCO). This yielded CTX F(ab')$_2$ (0.5 mL, 13.7 μM, 97% yield). Concentration was determined photometrically using ε_{280} = 140000 M^{-1}·cm^{-1}. Immobilized papain (0.5 mL, 0.25 mg·mL^{-1}) was activated with 10 mM DTT in digest buffer whilst shaking (1100 rpm) for 1 h at 37 °C. The resin was washed with digest buffer (without DTT) four times and CTX F(ab')$_2$ was added (0.5 mL, 13.7 μM). The mixture was incubated for 20 h at 37 °C whilst shaking (1100 rpm). Then the resin was separated from the digest using a filter column, and washed with BBS (25 mM sodium borate, 25 mM NaCl, 0.5 mM EDTA, pH 8.0) three times. The digest was combined with the washes and the buffer was exchanged completely for BBS using diafiltration columns (4 × 15 mL, GE Healthcare, 10000 MWCO) to remove impurities. This yielded CTX F(ab) (0.25 mL, 30 μM, 54% yield) as confirmed by SDS-PAGE and LCMS. Concentration was determined photometrically using ε_{280} = 70000 M^{-1}·cm^{-1}. Observed mass 49786 Da.

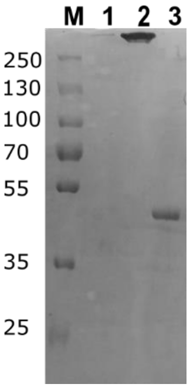

Figure S15: SDS-PAGE gel. M) Molecular weight marker in KDa; 1) Empty; 2) CTX; 3) CTX F(ab).

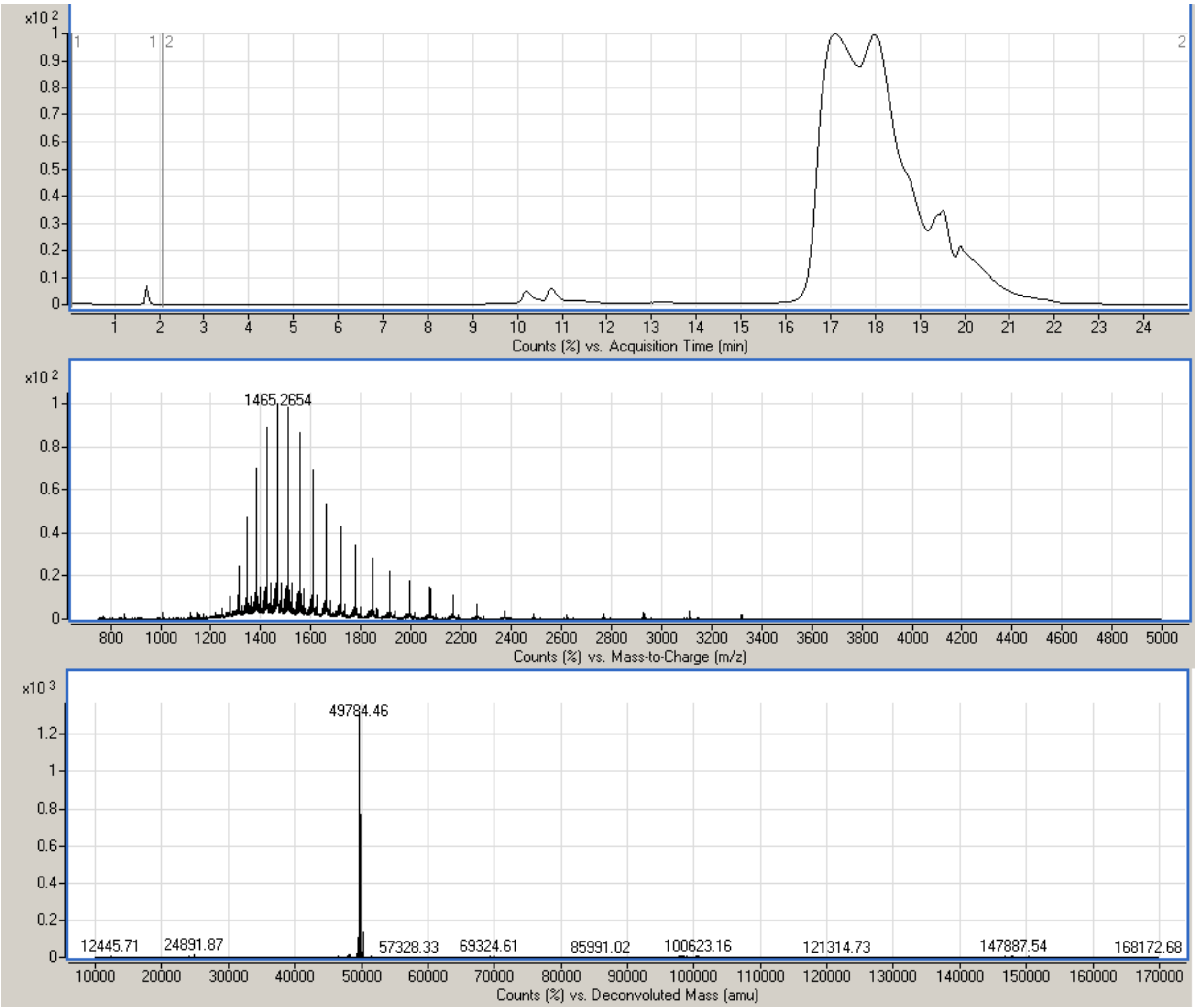

Figure S16: TIC, non-deconvoluted and deconvoluted MS data for CTX F(ab).

CTX F(ab) [disulfide]

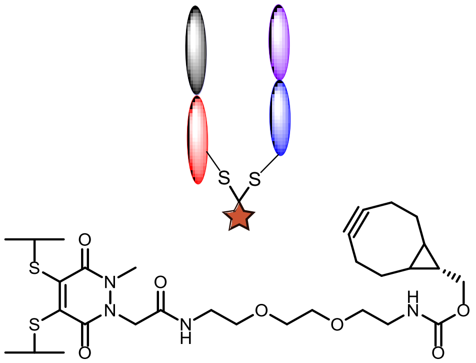

TCEP.HCl (2.5 µL, 20 mM in deionised water, 5 eq.) was added to a solution of CTX F(ab) **x** (250 µL, 20 µM) in BBS (25 mM sodium borate, 25 mM NaCl, 0.5 mM EDTA, 2% DMSO, pH 8.0) which had been pre-treated with pyridazinedione **3** (5.0 µL, 20 mM in DMSO, 10 eq.) and stored at 4 °C for 1 h previously. The reaction mixture was then stored at 4 °C for 15 h. Excess reagents were removed by repeated diafiltration into deionised water using VivaSpin sample concentrators (GE Healthcare, 10,000 MWCO). The samples were analysed by SDS-PAGE gel and LCMS. Observed mass 50273, expected mass 50275.

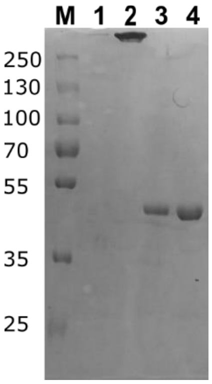

Figure S17: SDS-PAGE gel. M) Molecular weight marker in KDa; 1) Empty; 2) CTX; 3) CTX F(ab); 4) CTX F(ab) [disulfide].

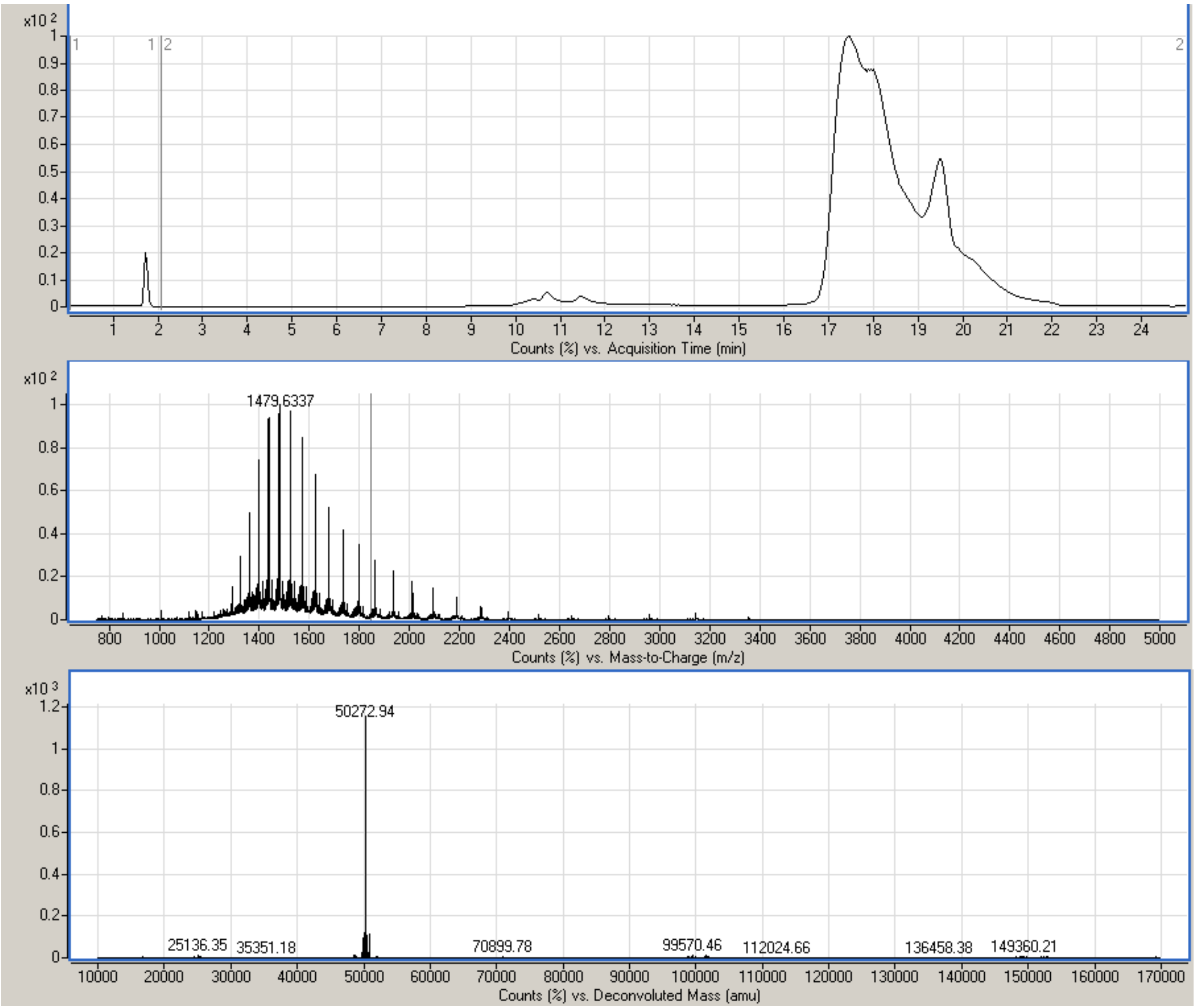

Figure S18: TIC, non-deconvoluted and deconvoluted MS data for CTX F(ab) [disulfide].

Nanoparticle stability

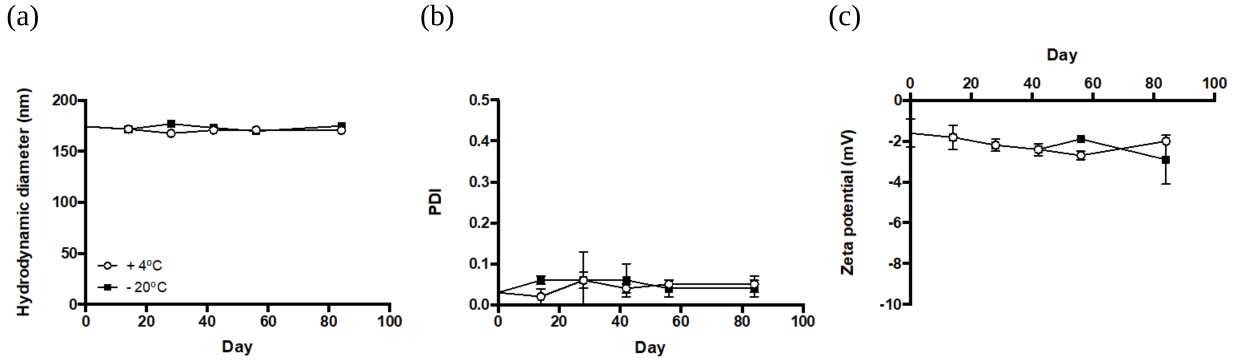

Figure S19: Stability assessment of nude azide NP. Aliquots of nude azide NP were stored at + 4 °C or – 20 °C. Nanoparticle (a) hydrodynamic diameter, (b) PDI and (c) zeta potential were measured at various timepoints post-formulation, ranging from 14 to 84 days. Data expressed as mean ± SD.

Cell surface expression of HER2

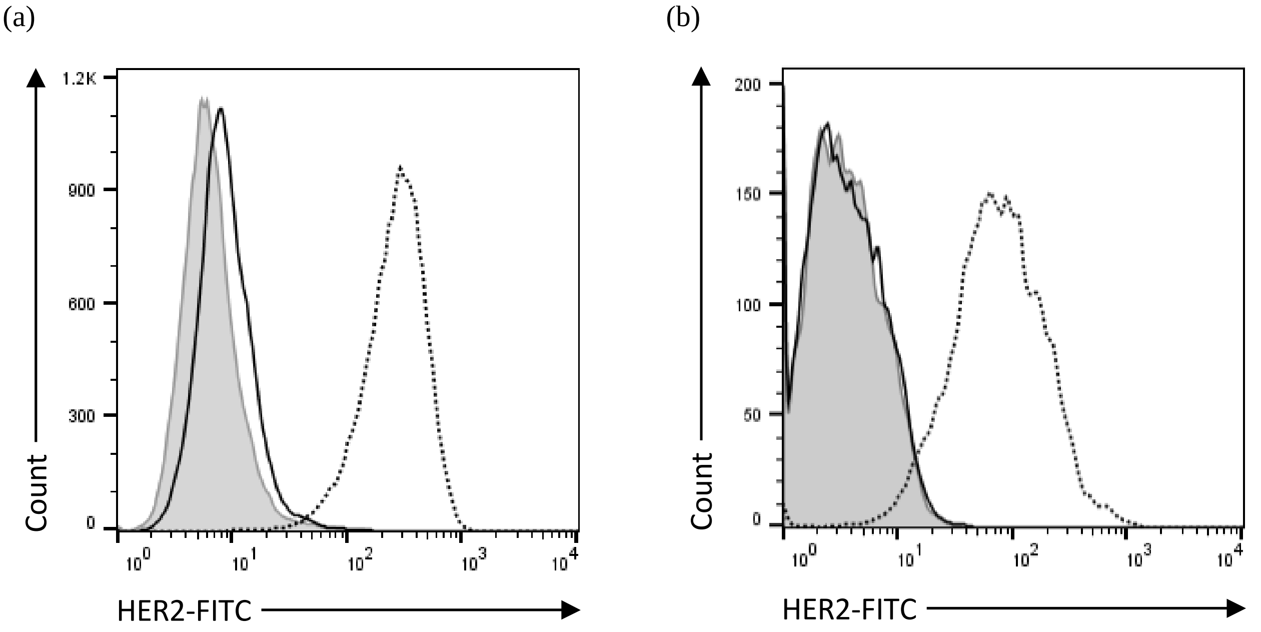

Figure S20: Confirmation of HER2 expression on breast cancer cell lines. HER2 expression on (a) HCC1954 and (b) BT474 cells was analysed by flow cytometry. Filled histograms (grey) represent unstained cells, whilst open histograms show cells stained with FITC-labelled isotype (solid line) or HER2 (dotted line) antibodies.

EGFR binding study

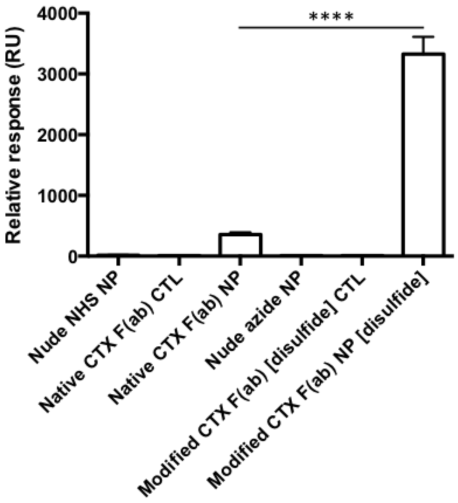

Figure S21: Translatability of site-specific conjugation approach to other antibody platforms. EGFR binding activity of native CTX F(ab) NP, modified CTX F(ab) NP [disulfide] and associated controls (10 mg polymer/mL) was assessed by SPR. An additional control (CTL) was included to confirm that the observed SPR responses were not attributed to residual unbound F(ab) within the nanoformulations. This involved incubating the F(ab) fragments with conjugation buffer devoid of nanoparticles and subjecting the samples to identical purification steps as for nanoparticles thereafter. Negligible responses were observed, confirming that the purification process was sufficient for complete removal of unbound F(ab) following the conjugation reactions. Data expressed as mean ± SEM. Statistical significance was established by one-way ANOVA and Tukey's post-hoc test (****$p \leq 0.0001$).

ELISA(s) for Traztuzumab Fab conjugate

A 96-well Maxisorp plate was coated overnight at 4 °C with HER2 (0.25 $\mu g \cdot mL^{-1}$ in 50 mM sodium carbonate buffer pH = 9.6, 100 μL). As a negative control one row was coated with only buffer. The solutions were removed and each well washed (2 × PBS with 0.05% Tween). The wells were subsequently coated with a 1% BSA solution in PBS for one hour at room temperature. After this the wells were emptied and washed (4 × PBS with 0.05% Tween). Solutions of TRAZ F(ab) **4**, TRAZ F(ab) [disulfide] **5**, and TRAZ F(ab) [disulfide]-Alexafluor488® **6** in PBS pH = 7.4 were prepared in the following dilutions: 60.0 nM, 15.0 nM, 3.75 nM, 0.938 nM, 0.234 nM and 0.00586 nM. The dilutions were placed into the wells, each in triplicate, and incubated for two hours at room temperature. As negative controls sodium carbonate buffer only and the antibodies at 60 nM in the absence of HER2 were also subjected to the same protocol. The solutions were removed and the wells washed (4 × PBS with 0.05% Tween). Detection antibody (100 μl of anti-human IgG, Fab-specific-HRP solution, 4 μL diluted in 20 mL of PBS) was added and left for one hour at room temperature. The solutions were removed and the wells washed (4 × PBS with 0.05% Tween). Finally, a TMB solution (1X TMB Solution, eBioscience, 100 μL) was added to each well. After five minutes the reaction was stopped through addition of 0.2 M sulfuric acid (50 μL). Absorbance was measured at 450 nm and corrected by subtracting the average of negative controls.

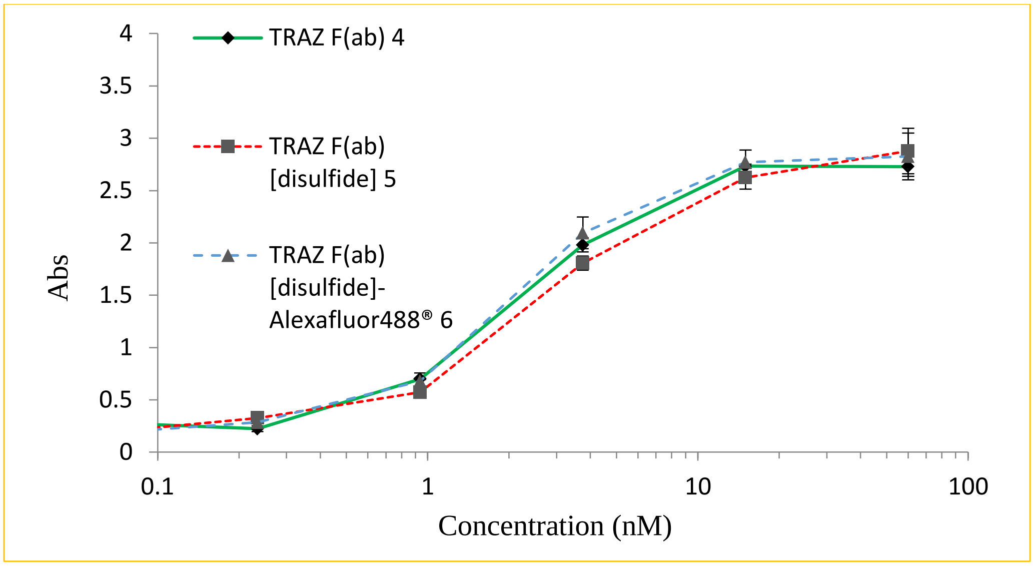

Figure S22: Binding activity of TRAZ F(ab) **4**, TRAZ F(ab) [disulfide] **5**, and TRAZ F(ab) [disulfide]-Alexafluor488® **6** by ELISA.

References

1 K. Hasumi *et al.*, *Bioorg. Med. Chem.*, 2014, **22**, 4162–4176.

2 J. Schmitz *et al.*, *ACS Med. Chem. Lett.*, 2014, **5**, 1076–1081.

3 A. Maruani *et al.*, *Chem. Commun.*, 2015, **51**, 15304–15307.
